# Comparative Simulations of Conformational Flexibility during Radical Transport in Ribonucleotide Reductase

**DOI:** 10.1021/acs.biochem.6c00487

**Published:** 2026-08-25

**Authors:** Matthew Tremblay, Sharon Hammes-Schiffer

**Affiliations:** Department of Chemistry, 6740 Princeton University, Princeton, New Jersey 08540, United States

## Abstract

Ribonucleotide reductase (RNR) is an essential enzyme that converts ribonucleotides to deoxyribonucleotides, utilizing a ∼32 Å chain of proton-coupled electron transfer (PCET) reactions spanning two protein subunits to generate a catalytic cysteine radical. Two cryogenic electron microscopy structures of the active complex of *E. coli* RNR are currently available. One structure was trapped in the preturnover state, prior to radical translocation, and the other structure was trapped in the midturnover state, with the radical in the active site. Herein, we use molecular dynamics simulations to investigate the differences in hydrogen-bonding interactions and conformational motions between the preturnover and midturnover states. Our simulations show that Y731, an interfacial tyrosine that participates in the PCET pathway, samples multiple conformations in both states, allowing it to participate in forward and reverse PCET between subunits. We also observe interfacial water channels between the protein subunits in both states. Moreover, our simulations show that E623, which is near Y730 in the preturnover structure and was shown by previous simulations to mediate PCET between Y731 and Y730, can also sample conformations distal to Y730 in the preturnover state, similar to its position in the midturnover structure. Our mixed quantum mechanical/molecular mechanical free energy simulations indicate that forward radical transfer from Y731 to Y730 is thermodynamically favorable with a reasonable free energy barrier, even in the absence of mediation by E623. These results provide insights into the critical role of conformational motions and flexibility in regulating PCET reactions at and near the protein subunit interface of RNR.

## Graphical abstract

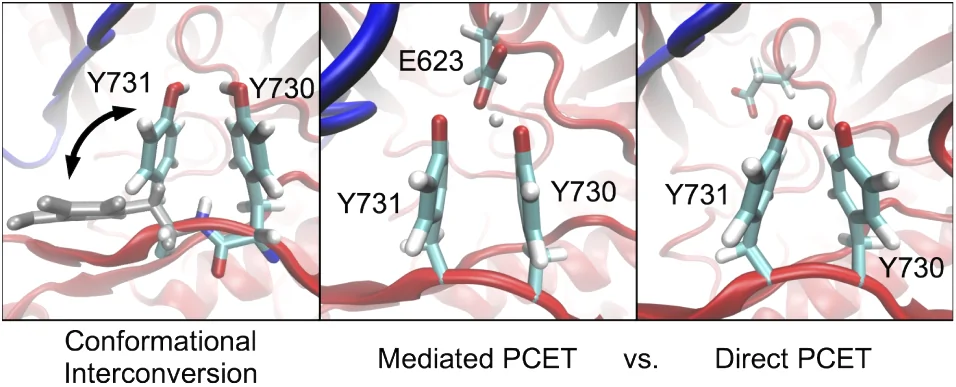

## Introduction

Ribonucleotide reductase (RNR) catalyzes the transformation of ribonucleotides into their corresponding deoxyribonucleotides, a vital process for DNA synthesis and repair in all organisms.^1^ This catalysis requires long-range radical transport that occurs through a series of proton-coupled electron transfer (PCET) reactions. Due to the importance of DNA biosynthesis for sustaining life, multiple inhibitors of RNR have been developed to treat cancer^2^ and antibiotic-resistant bacteria.^3^ Thus, understanding the mechanism of RNR, including long-range radical transport, is of biochemical and biomedical significance.

The active form of class Ia RNRs, such as those found in humans and *E. coli*, is a heterotetrameric complex (Figure 1) composed of two α subunits and two β subunits. Prior to the formation of the active complex, these subunits form homodimers denoted α_2_ and β_2_. Association of α_2_ with β_2_ leads to an asymmetric structure that contains a radical transport pathway connecting a tyrosyl radical (Y122^•^) in one β subunit to a cysteine (C439) in the active site buried in the interacting α subunit, where catalysis proceeds. After the thiyl radical is regenerated, reverse radical transport occurs along the same pathway. As is often the case in biological charge transfer, most of the electron transfer reactions that transport the radical are coupled to proton transfer reactions to avoid the creation of energetically unfavorable charged species,^4–6^ although recent computational work suggests that W48 in the β subunit may not transfer its proton upon reduction by Y356.^7^ The resulting radical transport pathway in RNR spans both protein subunits over a distance of ∼32 Å.

**Figure 1:**
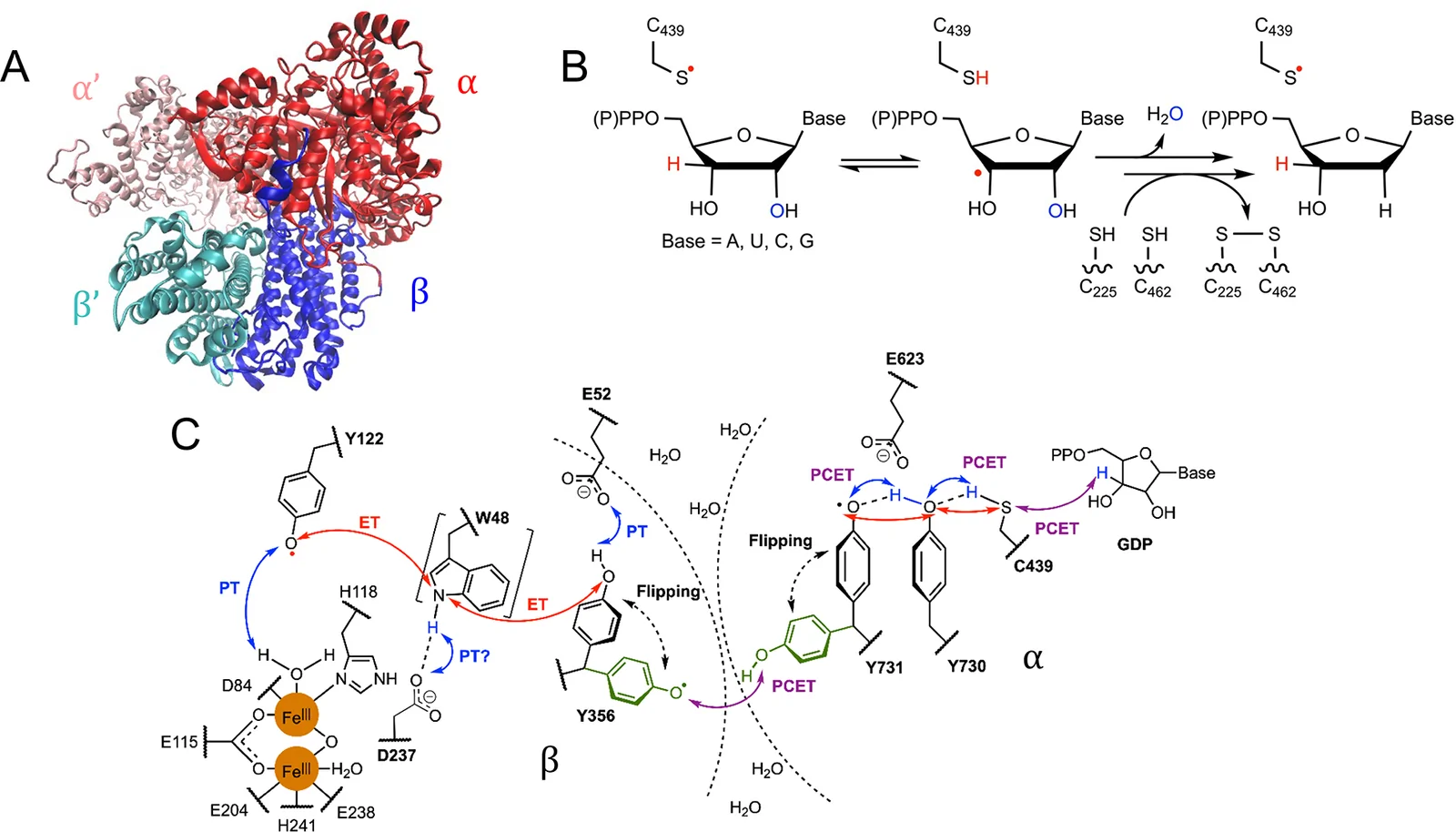
(A) The asymmetric structure of the tetrameric *E. coli* RNR. (B) The reduction of ribonucleotides to deoxyribonucleotides catalyzed by RNR. (C) The radical transfer pathway connecting Y122 (left) to the active site (right). The involvement of W48 is experimentally unconfirmed and thus is in square brackets. Note that the radical is on Y122 in the resting state, but for visualization purposes, we also show a radical on Y356 when it is flipped to perform PCET with Y731 and on Y731 when it is stacked to perform PCET with Y730. Proton transfer from Y356 to E52 has been proposed based on simulations,^7^ although other hypotheses have been proposed for this proton acceptor. Figure adapted from refs 7 and 8. Copyright 2026 National Academy of Sciences and Copyright 2020 American Chemical Society.

The interaction between the α_2_ and β_2_ homodimers is transient and tightly linked to the radical transport and catalysis processes.^9,10^ Due to its transient nature, the RNR active complex (α_2_β_2_) was not fully visualized until a cryogenic electron microscopy (cryo-EM) structure of *E. coli* RNR was published.^11^ This structure was captured using a double mutant β_2_ (E52Q/(2,3,5)-F_3_Y122-β_2_).^12^ The C-terminus of the β subunit had not been resolved in prior structures of the individual subunits of RNR and was shown to form noncovalent interactions with the α subunit, anchoring the subunits together and positioning Y356 in the middle of the PCET pathway. Biochemical analyses of this construct, combined with the asymmetric structure observed, indicate that one αβ pair, denoted α′ and β′, has turned over and released product to become “postturnover,” while the interacting pair, denoted α and β, is unable to initiate radical transport from Y122 due to the mutation and therefore remains “preturnover.”

Previous theoretical work from our group used this preturnover structure as a starting point and revealed key insights about the structure and mechanism of *E. coli* RNR. Y731, which was stacked against nearby Y730 in the cryo-EM structure, was able to flip out toward the β subunit,^8^ enabling it to engage in direct PCET with Y356.^13,14^ Additionally, E623, a glutamate residue near Y731 and Y730, was able to mediate PCET between these tyrosine residues by forming a hydrogen bond to Y730 and stabilizing the transferring proton as it transfers.^15^

More recently, an *E. coli* RNR cryo-EM structure that was trapped using 2′-azido-2′-deoxycytidine-5′-diphosphate (N_3_CDP) as an inhibitor of a wild-type active α_2_β_2_ complex was published.^16^ The azidonucleotide becomes covalently linked to C225 in the active site, prohibiting reverse radical transfer and product release. Spectroscopic^17^ and computational^18^ studies indicate that this complex has a radical trapped in the α active site on an adduct of the inhibitor and C225 and thus is trapped “midturnover.”

This new midturnover structure exhibits a number of intriguing features. Y731 was found to stack against Y730, as in the preturnover structure. However, the side chain of E623 has moved further away from these tyrosine residues, with its closest carboxylate oxygen 7.0 Å from the phenoxyl oxygen of Y730. In the preturnover structure, this distance is only 4.6 Å and was found to decrease during equilibration in molecular dynamics (MD) simulations, allowing it to mediate PCET between Y731 and Y730. Based on its location in the midturnover structure, however, E623 may not be close enough to be directly involved in the PCET reaction between Y730 and Y731. The midturnover structure was also solved at higher resolution than the preturnover structure (2.6 Å vs 3.6 Å), permitting direct visualization of trapped water. The water molecules at the α/β interface were observed to form two distinct water channels near Y356 and Y731. Water channels at the subunit interface have been proposed to provide water from bulk solvent to interact with Y356.^11^ Moreover, water has been proposed to potentially mediate the PCET reaction between Y356 and Y731 ^19^ and has been studied as a possible mediator of the PCET reaction between Y731 and Y730 in a model system.^20^

In this work, we utilize the two structures of the active RNR complex at distinct points in its turnover cycle to perform a comparison of the hydrogen-bonding interactions, conformational sampling, and free energy landscapes. Specifically, we present classical and mixed quantum mechanical/molecular mechanical (QM/MM) MD simulations based on these two structures. We interrogate the conformational motions and fluctuations of Y731, the water channels between the α and β subunits, and the hydrogen-bonding interactions that stabilize E623 further from the Y730 and Y731 tyrosine residues compared to its location in the preturnover structure. We also perform QM/MM free energy simulations^21^ of the PCET reaction between Y731 and Y730, highlighting both similarities and differences between the results based on the two structures. Forward radical transfer from Y731 to Y730 is shown to be thermodynamically favorable, with a reasonable free energy barrier, even in the absence of mediation by E623. This comparative study enhances our understanding of the roles of conformational changes and fluctuations in the mechanism of this biochemically essential enzyme.

## Methods

### Molecular Dynamics

Both the preturnover and midturnover cryo-EM structures were modified for the MD simulations. The preturnover cryo-EM structure (PDB 6W4X),^11^ which contains wild-type (WT) α_2_ and double mutant β_2_ (E52Q/2,3,5-F_3_Y122), was modified such that all residues were in their WT forms, as in our previous simulations of the preturnover state.^8^ No modifications were made to the guanosine-5′-diphosphate (GDP) substrate of the preturnover structure. The midturnover cryo-EM structure (PDB 9DB2)^16^ was trapped with WT α_2_β_2_ and the mechanistic inhibitor N_3_CDP. The inhibitor adduct in the α substrate binding site was modified to the native substrate cytidine-5′-diphosphate (CDP), and C225 was converted to regular cysteine. In the α′ substrate binding site, the single nitrogen atom was removed. Thus, the MD simulations for both structures were performed on WT proteins with native nucleoside diphosphate substrates. The location of the radical, substrate identity, and ligand on Fe_1_, which is closest to Y122, in each structure are given in Table 1. In both structures, Fe_2_ in the β and β′ metal centers is ligated by water, and the β′ metal center is in its *met* (inactive) state, with Y122 in its standard form as Y–OH and Fe_1_ ligated by water. Previous theoretical work has suggested that the dynamics in the α and β subunits are insensitive to the state of the β′ metal center.^8^ Unless otherwise specified, all residues are in their native forms.

**Table 1:** Radical Location, Substrate, and Fe_1_ Ligand for Classical MD Simulations Based on the Two RNR Structures

Structure	Radical location	Substrate	β Fe_1_ Ligand	β′ Fe_1_ Ligand
Preturnover	Y122	GDP	H_2_O	H_2_O
Midturnover	C439	CDP	OH^–^	H_2_O

These conditions represent the preturnover structure before radical translocation from Y122^•^ in the β subunit and the midturnover structure immediately prior to abstraction of hydrogen from the substrate by C439^•^. For both structures, protons were added using the H++ web server,^22^ and we manually adjusted the protonation states of the residues near the di-iron centers. Parameters for the iron centers, nucleotides, and Y122 radical in the preturnover structure were adapted from previous work,^8^ and those for the C439 radical were generated from the RESP procedure^23^ and are given in the Supporting Information. The protein residues and water were treated with the ff19SB^24^ and OPC^25^ force fields, respectively. The proteins were solvated with at least 15 Å of water on each side, and Na^+^ and Cl^–^ ions were added to neutralize any net charge and to create a salt concentration of ∼150 mM.

The structures were energy minimized, heated, and then equilibrated for 20 ns in the NPT ensemble and another 20 ns in the NVT ensemble. Three independent 300 ns trajectories were propagated for each structure in the NVT ensemble; all three trajectories originated from the same equilibrated geometry but were assigned different initial velocities. A 1 fs time step was used for the MD, and configurations were saved every 10 ps, totaling 90,000 frames for each starting structure. Long-range electrostatics were treated with the particle mesh Ewald algorithm^26^ with a cutoff of 10 Å. The systems were prepared with tleap,^27^ and all dynamics were propagated with Amber24.^28^ The trajectories were visualized with VMD^29^ and analyzed with CPPTRAJ^30^ and MDAnalysis.^31,32^ For the hydrogen-bond analyses, the heavy-atom distance and donor-hydrogen-acceptor angle cutoffs were 3.2 Å and 135°, respectively. Additional details about the classical MD simulations are provided in the Supporting Information.

### Umbrella Sampling

Umbrella sampling simulations^33^ were performed to compare the relative free energies of different Y731 conformations in the preturnover and midturnover states. A free energy profile was generated with respect to the χ_1_ dihedral angle (N–C_α_–C_β_–C_γ_), which has been previously used to characterize the flipping motion of Y731.^8^ These simulations were initiated from the geometries following NVT equilibration for the corresponding state; in both cases, the initial geometries had Y731 stacked against Y730 (χ_1_ ≈ 300°). Alan Grossfield’s implementation^34^ of the weighted histogram analysis method (WHAM) algorithm^35^ was used to generate the free energy profile. Further details on umbrella sampling are provided in the Supporting Information.

### Water Channel Analyses

Voronoi tessellation^36^ was used to probe the water channels identified by Westmoreland et al.^16^ Given a set of points, this algorithm divides all of space into cells, denoted tesserae, each consisting of the region closer to a particular point than to any other point. Here, the points are the atomic positions of the conformations sampled in the MD trajectories. If two atoms in different molecules have tesserae that share a face, then those molecules are defined as neighbors. This algorithm represents a rigorous method for defining molecular contacts that does not rely on arbitrary, distance-based criteria. Our group previously used Voronoi tessellation to define the first hydration shells of biomolecules for use in chiral-specific spectroscopy simulations.^37^

Here, we examine the hydration shells of the polar side chains of E52 in the β subunit and R639 in the α subunit, which lie on opposite sides of the α/β interface. These residues were selected for this analysis because they are both charged, thereby likely to make direct contact with water the majority of the time, and were identified in ref 16 as contacting the two identified water channels. The “yellow” water channel (see ref 16 and Figure 3 of this work) was defined as the set of water molecules that make contact with the carboxylate oxygens of E52, either directly or through other water molecules. To build this set for a given conformation, the first hydration shell was defined as all water molecules containing an atom that shares a tessera face with one of those carboxylate oxygen atoms (i.e., neighboring one of those two carboxylate oxygens). The second hydration shell was defined as all water molecules that are neighbors of the first hydration shell, and so forth. This procedure was repeated for the “pink” water channel (see ref 16 and Figure 3 of this work), using the five polar hydrogens of the guanidinium group of R639 instead of the carboxylate oxygens. The iterative process of building hydration shells was repeated until one or more water molecules were shared between the two water channels, indicating that the two channels intersected. This intersection could occur inside the α/β interface, revealing that the two channels are connected through the α/β interface, or it could occur in bulk water, indicating that the water channels are disconnected, as posited in ref 16. Identification of the hydration shells was performed with Python code developed in ref 38, which uses the freud library^39^ to access VORO++.^40^

### QM/MM Free Energy Simulations

The QM/MM finite temperature string method with umbrella sampling^41,42^ was used to compute the minimum free energy path (MFEP) as well as the reaction free energy and free energy barrier for the PCET reaction between Y731 and Y730. This iterative string method starts with an initial string represented by images connecting the reactant to the product, where each image is associated with values for the specified reaction coordinates. For each iteration, the QM/MM dynamics are propagated with harmonic restraints on the reaction coordinates to sample the corresponding region of conformational space. The centers for the restraints in the subsequent iteration are obtained by fitting a curve to the average values of the reaction coordinates from the current iteration and redistributing the images on that curve. This procedure is repeated until convergence.

We performed five different string simulations of forward radical transfer from Y731 to Y730. Four of these string simulations were based on the midturnover structure, considering direct, water-mediated, and through-water mechanisms in the α subunit and the direct mechanism in the α′ subunit, and the fifth string was based on the preturnover structure, considering the direct mechanism in the α subunit. In all of these simulations, the reactant was Y731–O^•^ and Y730–OH, and the product was Y731–OH and Y730–O^•^. The QM region was treated at the 6-31+G**/ωB97X-D^43,44^ level of theory. This level of theory was previously found to agree well with benchmark multireference wave function methods.^15^ The QM region for all five strings included the backbone and side chains of Y731 and Y730, and a water molecule was included in two of the strings. The MM region was described with the force field used for the classical MD described above. The dynamics were performed using the Amber/Q-Chem 6.0 ^45^ interface.^46^ The free energy surfaces were generated using the binless WHAM algorithm^47^ based on all the collected data for each string. Further details on the string simulations are provided in the Supporting Information.

## Results and Discussion

### Y731 Motion

In this section, we compare the conformations sampled by Y731 in classical MD simulations starting with the preturnover and midturnover structures. Three distinct conformations of Y731 have been observed^8^ through both biased and unbiased sampling (Figure 2A). These conformations are characterized by the χ_1_ side chain dihedral angle (N–C_α_–C_β_–C_γ_) of Y731. The angles χ_1_ ≈ 300°, 200°, and 70° represent conformations where Y731 is stacked against Y730, flipped out across the α/β interface toward Y356, and in an off-pathway geometry, respectively. The distributions of the Y731 χ_1_ dihedral angle in the unbiased MD simulations based on the preturnover and midturnover structures are shown in Figure 2B. To further probe the free energy landscape of these conformational changes, we performed one-dimensional umbrella sampling simulations. The resulting free energy curves with respect to χ_1_ are shown in Figure 2C.

**Figure 2:**
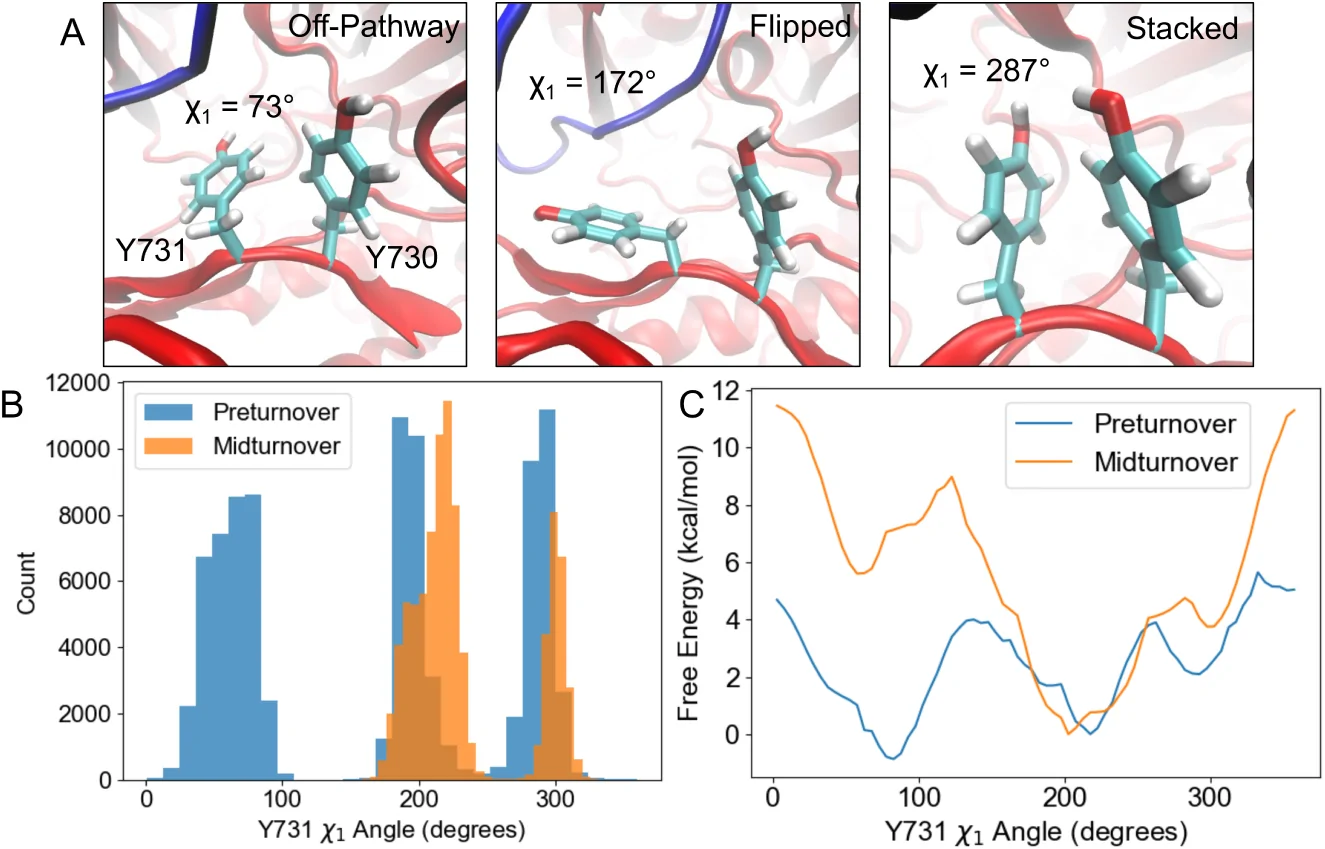
(A) Representative structures of the off-pathway, flipped, and stacked conformations. Geometries were selected from the unbiased simulations of the preturnover state. In each conformation, Y731 is shown on the left and Y730 on the right, with the α subunit in red and the β subunit in blue. (B) Distribution of the Y731 χ_1_ dihedral angle obtained from unbiased MD simulations based on the preturnover structure (blue) and midturnover structure (orange). Results from the three trajectories of each structure were combined, totaling 900 ns each. (C) Free energy profile with respect to the Y731 χ_1_ dihedral angle obtained from umbrella sampling simulations based on the preturnover structure (blue) and midturnover structure (orange). The free energy curves are aligned such that the flipped local minima are at 0 kcal/mol for both structures.

Spectroscopic experimental measurements have provided evidence for several different conformations of Y731 and their interconversion on the ns to μs time scale.^48–50^ Previous theoretical work suggested that this conformational flexibility of Y731 is key to its function at the subunit interface, as it can be poised to perform direct PCET with both Y356 when flipped out and with Y730 when stacked.^8,13,14^ The biological role of the off-pathway conformation is unclear. It has been observed in previous unbiased MD simulations based on the preturnover structure,^13^ although not in MD simulations with the radical on Y122.^8^ The previous simulations were performed with an earlier version of the Amber protein force field and a different water model (i.e., the ff14SB^51^ force field and the TIP3P water model^52^). These differences presumably contribute to the differences in the results presented in that previous work compared to the preturnover results presented herein. However, the general conclusions given below are the same for both force fields.

These data indicate that the three different conformations of Y731 are all readily accessible at room temperature. For both the preturnover and midturnover simulations, the flipped conformation is slightly favored over the stacked conformation, but these two conformations are connected by a low free energy barrier of <5 kcal/mol, enabling fast interconversion that is much faster than the RNR turnover time scale of several seconds. The off-pathway conformation is more disfavored in the midturnover state than in the preturnover state. This finding is consistent between the unbiased MD and the umbrella sampling MD, where the off-pathway conformation can only be accessed via a relatively high free energy barrier from the flipped or stacked conformation. Notably, the stacked conformation, which was observed in both of the cryo-EM structures,^11,16^ is not the lowest free energy minimum for the simulations based on either structure. The apparent preference for stacking in the cryo-EM structures could be the result of the radical trapping mechanisms used to collect the data for these structures. Alternatively, the preference for flipping in the free energy simulations may be due to the lack of stabilizing π-stacking interactions between the tyrosine side chains that are only approximately captured with the classical force field. The most important conclusion is that the simulations based on the preturnover and midturnover structures give very similar results regarding the Y731 conformational sampling between the flipped and stacked conformations.

### Water Channels at the α/β Interface

Analysis of the midturnover cryo-EM structure suggested that water channels on either side of the α/β interface are nonintersecting. These nonintersecting channels have been proposed to regulate the conformations of Y356 in the β subunit by providing distinct regions allowing hydrogen bonding of Y356 to water.^16^ To probe this hypothesis, we used Voronoi tessellation to identify the presence or absence of a through-water connection between the water channels located on opposite sides of the interface (Figure 3). The water channels were connected 95% of the time in the MD simulations based on the preturnover structure, whereas they were connected only 51% of the time in the MD simulations based on the midturnover structure. These water channels have been proposed to participate in the radical transfer from W48 to Y356 as the means for transporting the proton from Y356 to bulk solvent.^16,19,53,54^ Recent simulations have suggested that the Y356 proton may transfer to E52, which may then transfer its proton to water.^7^ Fluctuations in the connectivity of the two water channels could potentially play a role in regulating access of water to the interface and controlling the conformational motions of Y356. The observation of a connected water channel through the subunit interface for a substantial portion of the MD simulations based on both structures suggests that these water channels may not be responsible for the conformational preferences of Y356 throughout the turnover cycle.

**Figure 3:**
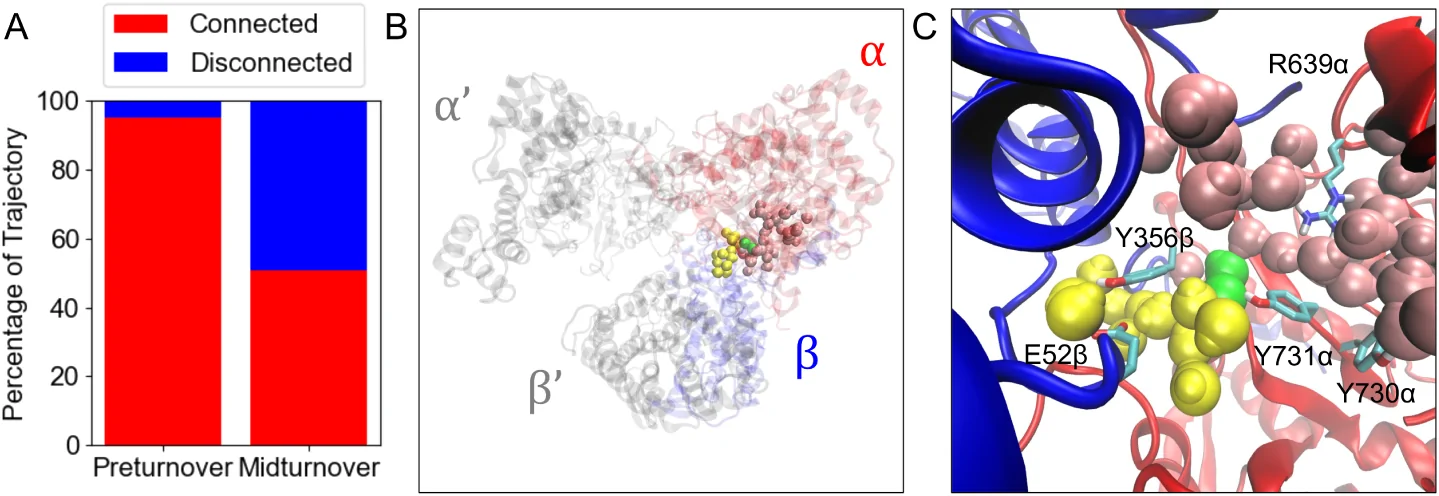
(A) Probabilities of the two water channels being connected through the α/β interface for the unbiased MD simulations based on the preturnover and midturnover structures, as determined from 900 ns of sampling for each case. (B, C) Water channel analysis for a representative conformation obtained from the MD simulations based on the midturnover structure, showing a single connected water channel. The hydration shells of E52 and R639 are shown in yellow and pink, respectively. The two water molecules that belong to the third hydration shell of both residues are shown in green and lie within the α/β interface, indicating that the water channel is connected for this conformation. The position of the water channel with respect to the entire protein structure is shown in panel (B). A magnified view of the water channel is shown in panel (C), illustrating that the hydration shells of E52 and R639 intersect in the middle of the interface, adjacent to Y731. A portion of the β subunit backbone has been omitted for visual clarity.

### Hydrogen Bonding in the α Subunit

One of the notable features of the midturnover cryo-EM structure is that E623 is located further from Y731 and Y730 than was observed in the preturnover structure. Specifically, the distance between the E623 carboxylate oxygen and the Y730 phenoxyl oxygen in the α subunit is 4.6 Å in the preturnover structure and 7.0 Å in the midturnover structure. We analyzed the hydrogen-bonding interactions of E623 in the latter environment, where it can no longer hydrogen bond to Y730 or Y731. Our MD simulations indicate that E623 is stable in this position over the entire trajectory (see Figure S3B). E623 is stabilized by accepting a hydrogen bond from the side chain of Q345 (Figure 4B and Table 2) and from water connected to the channel(s) between the α and β subunits.

**Figure 4:**
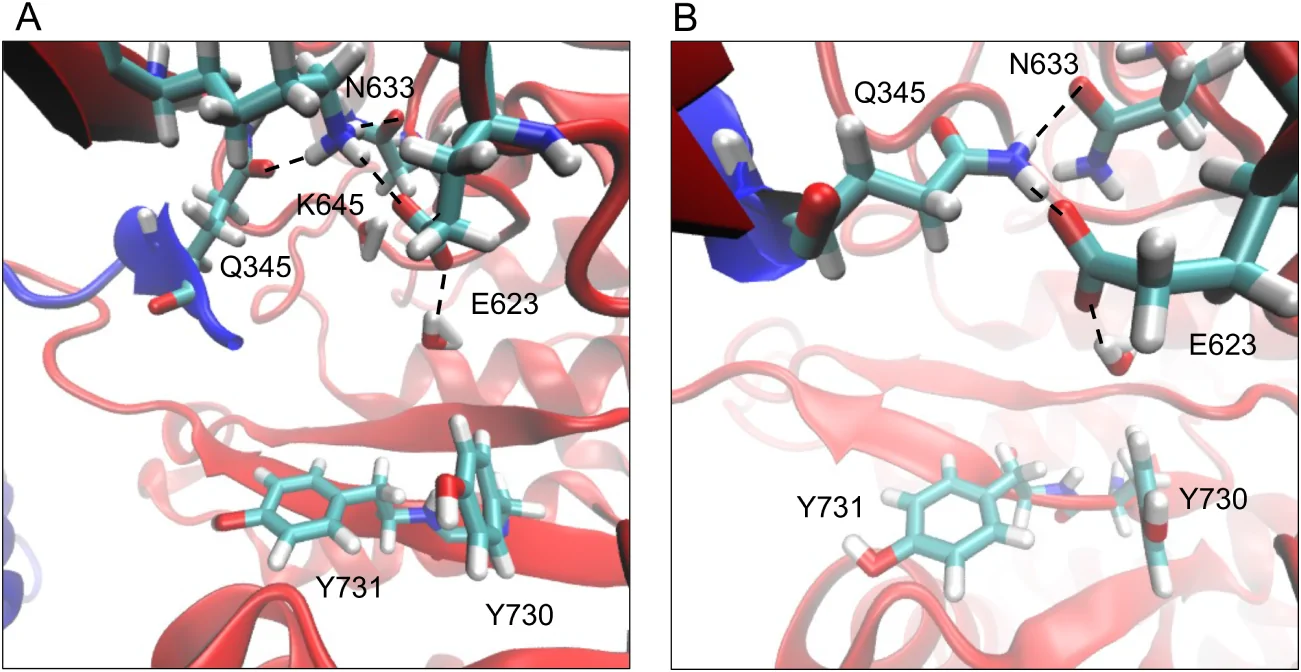
Hydrogen-bonding interactions of E623 when it is not interacting with Y731 or Y730 for representative conformations obtained from unbiased MD simulations based on the (A) preturnover structure and (B) midturnover structure. Hydrogen bonds are indicated with dashed lines. The backbones of the α and β subunits are shown in red and blue, respectively. Both conformations exhibit a hydrogen bond between E623 and water. The conformation in panel (A) shows K645 donating hydrogen bonds to E623, N633, and Q345, whereas the conformation in panel (B) shows Q345 donating hydrogen bonds to E623 and N633.

**Table 2:** Hydrogen-Bonding Partners for α Subunit Residue Side Chains

Donor	Y731	Y730	K645	K645	K645	Q345	Q345	H_2_O	Q345
Acceptor	E623	E623	E623	Q345	N633	E623	N633	E623	H_2_O
Preturnover^a^	36%	25%	80%	43%	45%	<1%	2%	86%	79%
Midturnover^a^	N.D.	N.D.	N.D.	25%	8%	77%	81%	100%	11%

a Percentage of frames in which at least one hydrogen bond is observed between the donor and acceptor. The sum of the percentages of frames in which E623 accepts a hydrogen bond is more than 100% because it is able to accept multiple hydrogen bonds simultaneously. “N.D.” indicates that the hydrogen bond is not observed at all within our sampling.

Furthermore, in all three independent MD trajectories based on the preturnover structure, E623 began to drift away from Y730 within 150 ns (Figure S3A). An example of how this repositioning can occur is shown in Figure S5. Following equilibration, E623 is hydrogen-bonded to Y730 (Figure S5A). Thermal fluctuations of the protein can lead to breaking of this hydrogen bond (Figure S5B). Once this hydrogen bond is broken, E623 can reorient its carboxylate group toward the α/β subunit interface, where it can form hydrogen bonds to water and the polar residues there. This reorientation occurs through rotations about the C–C bonds in the side chain.

Once E623 has shifted to this new position, it can form hydrogen-bonding interactions similar to those observed in the simulations originating from the midturnover structure. It accepts a hydrogen bond from K645, which simultaneously donates to Q345 and N633 (Figure 4A). Thus, E623 participates in similar but not identical hydrogen-bonding networks in this distal position observed later in the MD trajectories. These three independent MD trajectories were started from the same equilibrated structure but with different initial velocities. To further analyze this movement of E623, we propagated three additional trajectories, where each one started from the preturnover cryo-EM structure followed by a completely independent equilibration procedure and then a 300 ns production trajectory. E623 moved away from Y730 during two of the equilibration procedures but remained hydrogen-bonded to Y730 for the entire other production trajectory (Figure S4B).

Although these simulations cannot quantify the relative stabilities of the two conformations in which E623 has been observed, they highlight the flexibility of E623. This flexibility could potentially play a role in PCET between Y730 and Y731 because E623 has been shown to mediate the proton transfer between these two tyrosine residues in previous QM/MM simulations based on the preturnover structure.^15^ Moreover, E623 has also been hypothesized to potentially deprotonate Y731 during its PCET reaction with Y356.^16^ All of these roles would benefit from the type of flexibility that we have observed in our simulations.

### PCET between Y731 and Y730

Previous theoretical work from our group suggested that E623 can mediate the PCET reaction between Y731 and Y730 by stabilizing the proton as it transfers.^15^ These previous QM/MM free energy simulations were based on the preturnover structure, in which the distance between the E623 carboxylate oxygen and the Y730 phenoxyl oxygen was only 4.6 Å and decreased to form a hydrogen bond during equilibration. As discussed in the previous subsection, the MD simulations based on the preturnover structure presented herein indicate that E623 also samples a stable conformation further from Y730, as found in the midturnover structure. To assess the impact of this new conformation on PCET between Y731 and Y730, we performed QM/MM free energy simulations based on the preturnover structure starting with a conformation in which E623 was located further from Y730 and Y731, thereby enabling the direct mechanism. In addition, we performed QM/MM free energy simulations based on the midturnover structure, considering the direct mechanism as well as two different mechanisms involving an intervening water molecule. Although these results use a different force field than the previous work, the results can still be compared at a qualitative level.

To investigate these possible mechanisms for PCET between Y731 and Y730, we performed five different finite temperature string simulations. In all cases, the reaction studied is forward radical transfer from Y731 to Y730: Y731^•^ + Y730 → Y731 + Y730^•^. For all five strings, the two tyrosine side chains remain stacked, and the radical is placed on Y731 for the reactant. Based on the preturnover structure, we investigated the direct mechanism, where E623 is in its conformation further from Y730. Based on the midturnover structure, we investigated the direct mechanism, the water-mediated mechanism, in which a bridging water remains hydrogen-bonded to the proton during PCET, and the through-water mechanism, in which the water transfers its proton to Y731 as it accepts a proton from Y730 in a concerted proton relay process. These three mechanisms, as well as the E623-mediated mechanism, are shown in Figure 5. As a control, we also simulated direct PCET between Y731 and Y730 in the α′ subunit based on the midturnover structure. The α′ subunit is viewed as a control because it is noninteracting and presumed to be postturnover. The reaction free energies and free energy barriers for these five strings, as well as those obtained previously for the E623-mediated mechanism based on the preturnover structure, are given in Table 3.

**Figure 5:**
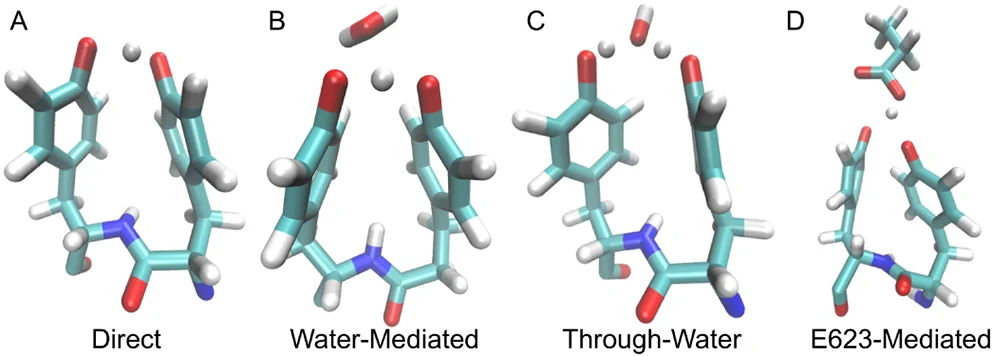
Representative conformations showing the mechanisms for PCET between Y731 and Y730 considered in this work: (A) direct PCET, (B) water-mediated PCET, (C) PCET through a bridging water, and (D) E623-mediated PCET. The conformations shown in A, B, and C were obtained from QM/MM free energy simulations based on the midturnover structure, and the conformation shown in D was obtained from QM/MM free energy simulations based on the preturnover structure.

**Table 3:** Reaction Free Energies and Free Energy Barriers for Forward Radical Transfer from Y731 to Y730

Structure	Mechanism	Δ*G* ^a^ (kcal/mol)	Δ*G* ^‡^ ^a^ (kcal/mol)
Midturnover	Direct	–1.4	5.1
Midturnover	Water-Mediated	–1.8	8.0
Midturnover	Through-Water	–0.6	11.6
Midturnover	α′	+1.1	9.4
Preturnover	Direct	–4.0	7.5
Preturnover	E623-Mediated^b^	–7.6	5.0

a Free energies are given for Y731^•^ + Y730 → Y731 + Y730^•^, and a negative Δ*G* value indicates that forward radical transfer is favorable.

b These data were obtained from ref 15 and were computed using the Amber ff14SB protein force field and the TIP3P water model.

These data suggest that forward radical transfer is possible without mediation by E623. All five strings for radical transfer from Y731 to Y730 within the α subunit are exoergic, although the previously studied E623-mediated reaction based on the preturnover structure remains the most thermodynamically favorable. These five strings also have reasonably low free energy barriers less than 12 kcal/mol, such that the PCET reaction will be much faster than the RNR turnover time scale of several seconds.

The direct mechanisms in the simulations based on the preturnover and midturnover structures are each exoergic with a similar free energy barrier, although their values of Δ*G* and Δ*G* ^‡^ differ slightly. The more negative Δ*G* for the direct mechanism based on the preturnover structure can be ascribed in part to the formation of a hydrogen bond between water and Y730^•^ in the product but not in the reactant. This hydrogen bond was not observed for the direct mechanism based on the midturnover structure, possibly due to limitations of conformational sampling. The higher free energy barrier for the direct mechanism based on the preturnover structure can be attributed in part to the greater O–O distance in the reactant geometry compared to this distance in the reactant geometry for the direct mechanism based on the midturnover structure. However, we note that the differences between Δ*G* and Δ*G* ^‡^ for the direct mechanism based on the preturnover and midturnover structures are considered to be within the uncertainty of the computational methodology, particularly given the limitations in conformational sampling.

Mediation by water does not significantly impact the exoergicity relative to the direct mechanism but increases the free energy barrier. The relatively similar values of Δ*G* and Δ*G* ^‡^ for the direct and water-mediated mechanisms suggest that they could compete, depending on the hydrogen-bonding configurations of Y731 and Y730 when Y731 becomes a radical and stacks with Y730. The higher barrier and less favorable Δ*G* of the through-water mechanism suggest that this mechanism is less likely.

In simulations based on both structures, Y731 can readily access stacked conformations allowing it to perform collinear PCET with Y730. Within these conformations, there are several hydrogen-bonded networks that each may promote a distinct mechanism for this PCET reaction (i.e., direct, water-mediated, or E623-mediated). Our results suggest that, despite the variety of possible conformations, the radical transfer reaction from Y731 to Y730 within the α subunit remains exoergic with a reasonably low free energy barrier. In contrast, radical transfer from Y731 to Y730 in the postturnover α′ subunit is endoergic for the strings based on both cryo-EM structures, favoring reverse PCET, as expected in the postturnover state. The free energy barrier for radical transfer in the α′ subunit calculated in this work is lower than that found in our previous work,^15^ although the direct mechanism was simulated in both cases. We ascribe this difference to a combination of different initial structures and limitations in conformational sampling, which are especially pronounced for the more mobile α′ subunit, as well as relatively minor differences in the force fields.

We note that these QM/MM free energy string simulations describe both the forward and reverse reactions between Y731 and Y730. The resulting MFEPs and free energy surfaces are valid for both the forward and reverse reactions. However, the results of the simulations depend on the initial structure because of limitations in conformational sampling. The preturnover structure likely favors forward radical transport, but the preferred direction for radical transport in the midturnover structure is less clear, as it was captured with the radical trapped within the active site after forward radical transport but before catalysis.

## Conclusion

Herein, we performed comparative simulations of *E. coli* RNR based on cryo-EM structures trapped at two different points in its turnover cycle. One cryo-EM structure was trapped in the preturnover state, with the radical presumed to be on Y122 prior to radical translocation, and the other cryo-EM structure was trapped in the midturnover state, with the radical presumed to be within the active site. Unbiased and umbrella sampling MD simulations showed that Y731 is able to sample multiple conformations at room temperature in both states. Specifically, Y731 samples a conformation in which it is stacked with Y730 and a conformation in which it is flipped out to extend across the interface between the α and β subunits. This conformational flexibility enables Y731 to participate in direct forward and reverse PCET with both Y356 in the β subunit and Y730 in the α subunit. Moreover, the water within the α/β interface constitutes one continuous water channel during the majority of the simulations, providing the possibility of proton transport through water networks, although two disconnected water channels are also observed during portions of the simulations, as observed in the midturnover cryo-EM structure.

An important distinction between the preturnover and midturnover cryo-EM structures is that E623, which was located near Y730 in the preturnover structure and was found to mediate PCET between Y731 and Y730 in previous simulations, is located more distal to Y730 in the midturnover structure. Our simulations based on the midturnover structure characterized the hydrogen-bonding interactions stabilizing E623 in this distal position. Additionally, our simulations based on the preturnover structure showed that E623 can also move away from Y730 and form stable hydrogen-bonding interactions in this distal location prior to radical translocation. This conformational flexibility of E623 may play an important functional role in radical transport. QM/MM free energy simulations of direct radical transfer from Y731 to Y730 revealed that this reaction is thermodynamically favorable with a reasonably low free energy barrier even when E623 is unable to serve as a mediator.

This work highlights the power of atomistic MD simulations for understanding conformational sampling and the resulting structural changes in proteins. Static cryo-EM snapshots provide only limited information about conformations sampled during catalysis, and MD simulations based on such snapshots provide useful additional information. However, due to the inherent sampling limitations associated with MD, particularly for a protein as large and flexible as RNR, the availability of multiple cryo-EM structures of the same protein at different points in the turnover cycle is imperative for capturing large-scale conformational changes. This synergy between experiment and theory is critical for advancing our understanding of the complex mechanism of catalysis in RNR and other biochemically essential enzymes.

## Supplementary Material





## Data Availability

The data for this work has been uploaded to a Zenodo repository (DOI: 10.5281/zenodo.21656213) and made publicly available.

## Notes

Parametrization of the protein complex; equilibration and dynamics procedures; QM/MM free energy strings (PDF)
Representative PDB and Amber structure files for the preturnover and midturnover RNR structures (ZIP)
