# Supplementary material for: Comparative Simulations of Conformational Flexibility during Radical Transport in Ribonucleotide Reductase
Source: Biochemistry. 2026 Aug 25;65(18):2896–904. doi: 10.1021/acs.biochem.6c00487 (PMC13585066; doi:10.1021/acs.biochem.6c00487)
Supplement: Supplementary file 1 [file bi6c00487_si_001.pdf]

Supporting Information
Comparative Simulations of Conformational Flexibility During Radical Transport in Ribonucleotide Reductase

Matthew Tremblay and Sharon Hammes-Schiffer*

Department of Chemistry, Princeton University, Princeton, NJ 08540

E-mail: shs566@princeton.edu

Contents

1 Parameterization and System Building **S3**

1.1 Preturnover Structure . . . S4

1.2 Midturnover Structure . . . S4

1.2.1 Cysteine Radical . . . S5

1.2.2 Bound Nucleotides . . . S5

2 Equilibration and Unbiased Molecular Dynamics **S6**

3 Classical Umbrella Sampling of the Y731 Dihedral Angle **S8**

4 E623 Distance Analysis **S9**

5 QM/MM String Simulations **S12**

5.1 Direct — Midturnover . . . S15

5.2 Water-Mediated — Midturnover . . . S15

5.3 Through-Water — Midturnover . . . S18

5.4 Postturnover α' — Midturnover . . . S19

5.5 Direct — Preturnover . . . S21

5.6 Convergence and Statistical Analyses . . . S22

References **S24**

1 Parameterization and System Building

The preturnover and midturnover structures were manually modified as described below and then processed with pdb4amber[1] before protons were added with H++.[2] Residues near the metal cofactors had their protonation states configured manually, as H++ does not account for metal ion coordination. The tleap program was used to build the Amber input coordinate and topology files, solvating each protein with a periodic rectilinear box of OPC water[3] extending at least 15 Å on each side of the protein. Na^+ and Cl^- ions were added to neutralize any net charge and then to create a salt concentration of ∼150 mM, based on the total box volume following solvation and prior to any equilibration. The sodium and chloride ions were treated with the 12-6 parameters for OPC water from Merz and coworkers.[4]

In contrast to our previous simulations of the preturnover structure,[5] water was described by the OPC force field, and protein residues were described by the Amber ff19SB force field[6] for all simulations herein. The parameters for the metal centers in both the preturnover and midturnover structures were obtained from previous work on the preturnover structure,[5] as the geometries of the metal centers in the two structures are similar. However, the atom type of the C_α atoms in the protein residues ligating the iron atoms was changed from CX to XC to use the ff19SB CMAP for backbone dihedrals. For each modified residue, the existing CMAP for the canonical version of that residue was used. A hybrid bonded/nonbonded scheme was used for the metal centers; in this scheme, the amino acids and the bridging oxygen coordinated to the iron atoms are treated with bonds, and the water and hydroxide are harmonically restrained to their respective metal ions. Note that because the parameters for the metal center and ligands were developed using the TIP3P water model,[7] the water molecules ligated to iron atoms in both structures are three-point models, unlike the four-point water molecules used for bulk solvent. The charges on these ligated water molecules were determined from the previous parameterization of the metal complex[5] and thus are not associated with any specific water model, and the oxygen atoms are ligated (with a harmonic restraint) to an iron atom. The details of the parameterization of the diiron complex are

unlikely to impact the simulations herein, which focus on a region far from the iron atoms.

1.1 Preturnover Structure

For the simulations based on the preturnover structure, the β' metal cofactor was assumed to be in the *met* state, with a standard (reduced) Y122 and one water ligated to each of the two Fe atoms. The β metal cofactor was assumed to be in the state immediately prior to radical translocation, with Y122 in its radical state and a water molecule ligated to each of Fe_1 and Fe_2. Here, Fe_1 is the iron atom closer to Y122. The partial charges for the Y122 tyrosyl radical were obtained from previous work.[5] The atom type of the C_α atom in the tyrosyl radical was changed to XC to use the ff19SB backbone CMAP.

As discussed in the main text, the deposited cryo-EM structure (PDB 6W4X)[8] was modified such that the two mutations (E52Q/2,3,5-F_3Y122) made to β_2 were reverted to their WT state. No modifications were made to the bound substrate and effectors.

1.2 Midturnover Structure

For the simulations based on the midturnover structure, the β' metal cofactor was also assumed to be in the *met* state. The β metal cofactor was assumed to be in the state after radical translocation, with a standard (reduced) Y122, a hydroxide ion ligated to Fe_1, and water ligated to Fe_2. The water molecule ligated to Fe_1 was presumed to have donated its proton to Y122 in the first step of radical translocation.

The cryo-EM structure (PDB 9DB2)[9] was manually modified to remove the residual N-centered radical in the α' active site and to convert the cysteine-inhibitor adduct in the α active site into regular cysteine and CDP. No modification was made to the bound effectors. All water present in the cryo-EM structure was retained in system building. Residues 341 to 360 of the β' subunit were not included in the cryo-EM structure due to their flexibility; residues 361 to 374 were included in the structure but were discarded during system preparation, as they would have been disconnected from the remainder of β'.

1.2.1 Cysteine Radical

Partial charges for the thiyl radical at C439 in the midturnover structure were derived from the RESP protocol.[10] First, tleap[1] was used to construct a cysteine residue capped with acetyl and N-methyl amine groups. Following removal of the thiol proton, GaussView 6[11] was used to create two initial geometries, corresponding to the backbone dihedral angles of cysteine in an α helix and in a β sheet.[12] Gaussian 16[13] was used to optimize the geometries of these structures at the Hartree-Fock 6-31G(d) level of theory and to calculate an electrostatic potential around each of them. Then, a single set of atomic partial charges was obtained using a least-squares fitting method to reproduce both of the electrostatic potentials simultaneously, while the backbone charges were constrained to the values used by all ff19SB protein residues. Bonded (including backbone CMAP) and Lennard-Jones parameters were taken from the standard cysteine ff19SB parameters. Table S1 contains the Amber atom type and RESP charge for each atom in the cysteine radical.

Table S1: Cysteine Radical Atom Names, Types, and Partial Charges

Atom Name	ff19SB Atom Type	Partial Charge
N	N	-0.415700
H	H	0.271900
CA	XC	-0.003776
HA	H1	0.099507
CB	2C	0.028104
HB2	H1	0.079187
HB3	H1	0.079187
SG	SH	-0.167809
C	C	0.597300
O	O	-0.567900

1.2.2 Bound Nucleotides

The midturnover structure contains bound substrate CDP, activity effector ATP, and specificity affector dATP. The charges for the phosphate tails were taken from the literature on polyphosphates,[14] and parmbsc1 charges[15] for the nucleobases and sugar were used. The

parmbsc1 force field was also used for all bonded and Lennard-Jones parameters of these species.

2 Equilibration and Unbiased Molecular Dynamics

Hereafter, the preturnover and midturnover structures were treated identically and thus will not be discussed separately. All classical simulations were performed with Amber 24.[16] Specifically, energy minimizations were performed with the MPI-accelerated version of pmemd, and molecular dynamics (MD) simulations were performed with the CUDA-accelerated version of pmemd. Unless otherwise specified, all MD simulations were performed with an integration timestep of 1 fs using Langevin dynamics with $\gamma = 2.0$ ps^{-1}. In all MD simulations, SETTLE was used to constrain the internal degrees of freedom of the solvent,[17] and SHAKE[18] was used to constrain all bonds involving hydrogen in the protein, nucleotides, and cofactor (solute) to their equilibrium length. The NPT MD used the Berendsen barostat set at 1.01325 bar. At each stage of MD (*i.e.*, heating, NPT equilibration, NVT equilibration, and NVT production), new randomized velocities were selected for each atom from a Boltzmann distribution. Long-range electrostatic interactions were treated with the particle-mesh Ewald algorithm[19] with a cutoff of 10 Å.

The system was minimized, heated, and equilibrated as detailed in Table S2. The time and temperature information for the heating steps are in Table S3. After NVT equilibration, three production MD trajectories were propagated from the same equilibrated structure starting with independent velocities obtained from a Boltzmann distribution. Each trajectory was propagated for 300 ns, with coordinates saved every 10 ps. In total, 90,000 frames were collected for each state (*i.e.*, the preturnover and midturnover states initiated from the preturnover and midturnover structures, respectively). The C_α root-mean square deviations (RMSDs) for the equilibration and production trajectories, as compared to their respective cryo-EM structures, are shown in Figure S1.

Table S2: Steps for Optimization and Equilibration of Solvated System

Step	Type[a]	Length[b]	Temperature (K)	Restraint Weight (kcal/mol/Å^2)[c]	Restraint Selection[d]
1	Min	5,000 SD	-	500	All solute
2	NVT	50 ps	300	500	All solute
3	NPT	1 ns	300	200	All solute
4	Min	2,000 SD, 3,000 CG	-	100	Solute, non-H
5	Min	2,000 SD, 3,000 CG	-	100	Solute backbone
6	Min	2,000 SD, 3,000 CG	-	50	Solute backbone
7	Min	2,000 SD, 3,000 CG	-	10	Solute backbone
8	Min	2,000 SD, 3,000 CG	-	-	-
9	NPT	360 ps	See Table S3	-	-
10	NPT	20 ns	300	-	-
11	NVT	20 ns	300	-	-

[a] Min = Minimization, NVT/NPT = MD within that ensemble.
[b] SD = steps of Steepest Descent energy minimization, CG = steps of Conjugate Gradient energy minimization.
[c] The restraint weights k are defined using Amber's convention of $U = k(x - x_0)^2$.
[d] Here, solute refers to protein, nucleotides, and metallocofactors.

Table S3: Heating Steps in the NPT Ensemble[a]

Step	Length (ps)	Final Temperature (K)
1	10	50
2	50	50
3	10	100
4	50	100
5	10	150
6	50	150
7	10	200
8	50	200
9	10	250
10	50	250
11	10	300
12	50	300

[a] The initial temperature for Step 1 was 0 K, and the initial temperature for each other step was the final temperature from the previous step.

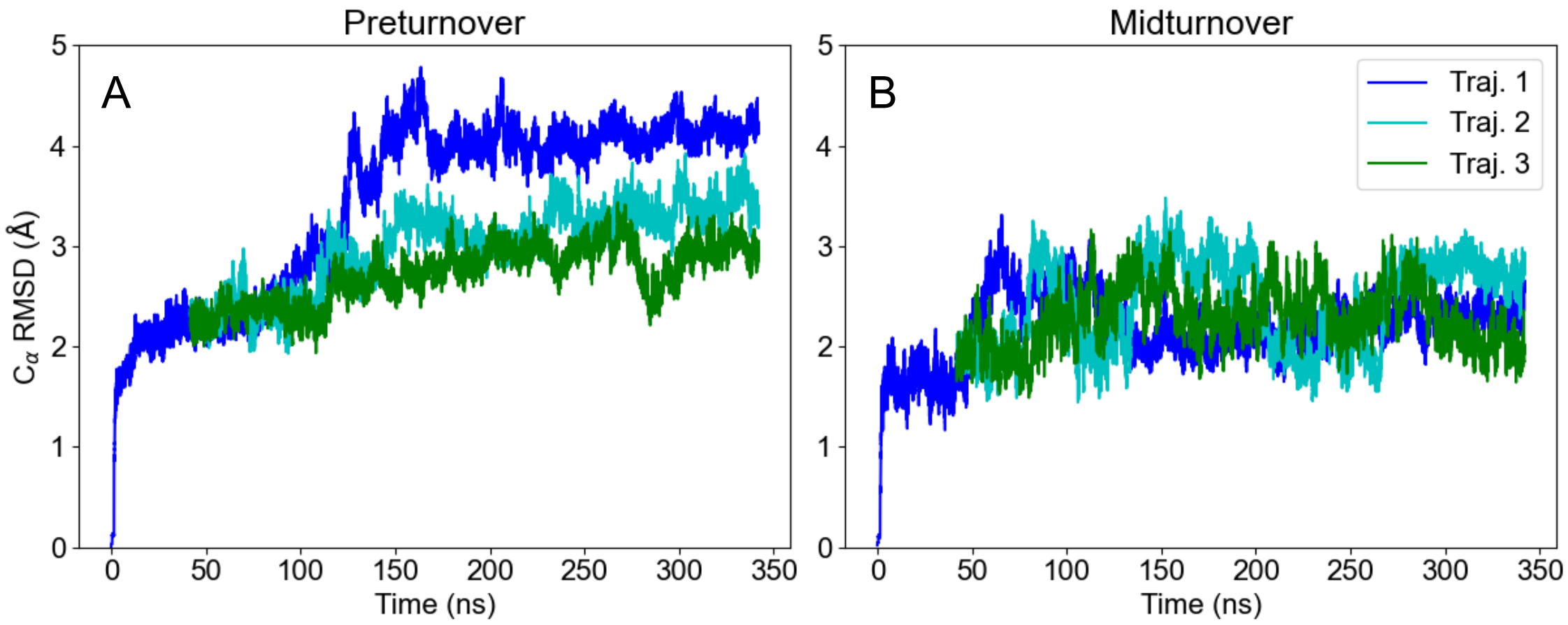

Figure S1: C_α RMSD for (A) the preturnover structure and (B) the midturnover structure during the equilibration and production MD trajectories. The three colors correspond to the three independent trajectories. The equilibration phase ended at 40 ns.

3 Classical Umbrella Sampling of the Y731 Dihedral Angle

The umbrella sampling simulations began from the NVT equilibrated structure, in which the Y731 χ_1 dihedral angle (N$-$C$_\alpha$$-C_\beta$$-C_\gamma$) was $\sim$300° for both the preturnover and midturnover structures. Each structure was further equilibrated for 20 ns with a harmonic restraint on χ_1 centered on 300°. All classical umbrella sampling in this work was performed at 300 K and used a force constant of k = 200 kcal/mol/radian^2, using Amber's convention of $U = k(\phi - \phi_0)^2$. The final geometry after 20 ns was used as the initial geometry for 20 ns equilibration trajectories with restraints centered on 270° and 330°. The final geometries from those equilibration steps were used to initiate equilibration trajectories with restraints at 240° and 360°, and so forth to obtain twelve equilibrated structures restrained to χ_1 values from 30° to 360° in 30° increments. These structures were each used to start five more trajectories to produce structures corresponding to 72 windows, spanning 360° in 5° increments, each equilibrated with their restraint for 20 ns. After this equilibration phase, we propagated 15 ns of production MD starting from each equilibrated structure, with χ_1 recorded every 10 fs. These values of χ_1 were used to create a periodic free energy profile for

the rotational motion of Y731 using Alan Grossfield's implementation of WHAM,[20,21] with a convergence criterion of 0.0001. The free energy profiles created by analyzing the first and last 7.5 ns of the production trajectories are shown in Figure S2, along with the free energy profile generated from the entire 15 ns data set. The reasonable agreement between the free energy profiles generated from the first and second halves of the data, as well as from the full data set, indicates that the umbrella sampling simulations are converged.

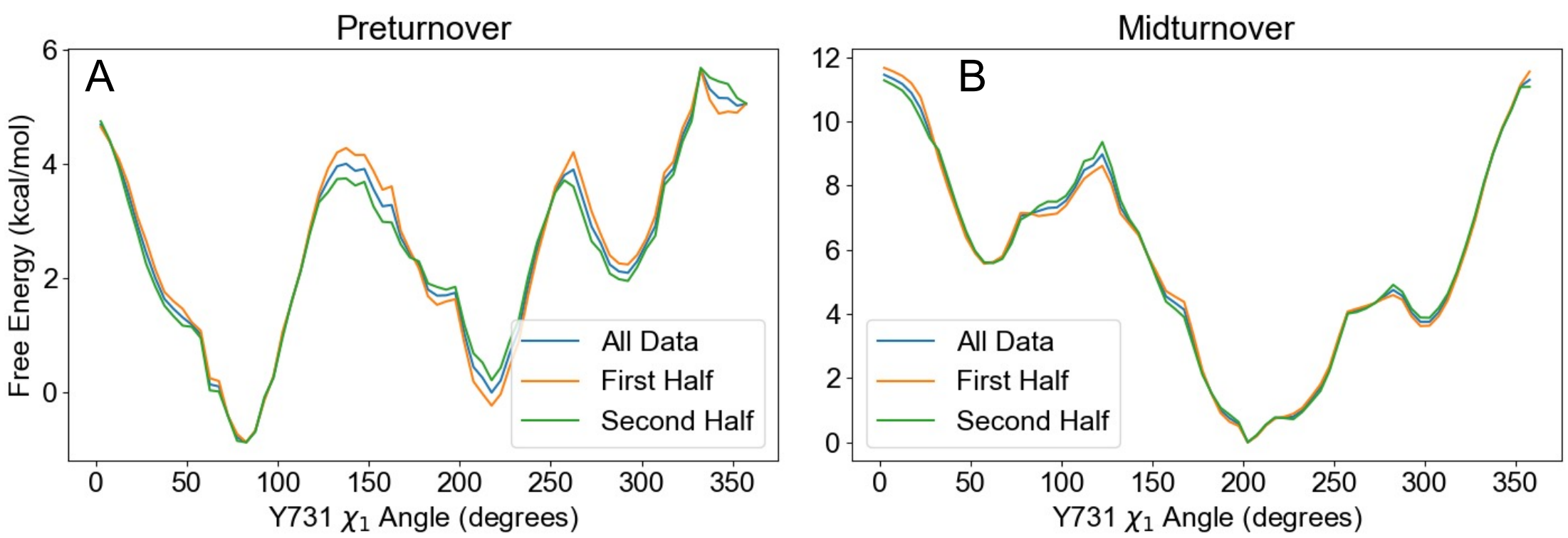

Figure S2: Convergence analysis for the free energy profiles with respect to the Y731 χ_1 dihedral angle in the umbrella sampling simulations based on (A) the preturnover structure and (B) the midturnover structure. The free energy profiles generated from the first and second halves of the data set, as well as from the full data set, are in reasonable agreement, indicating that these umbrella sampling simulations are converged.

4 E623 Distance Analysis

To determine whether the observed position of E623 in the midturnover and preturnover cryo-EM structures was stable during the MD simulations, the distance from the phenoxyl oxygen of Y730 to the closer of the two carboxylate oxygen atoms of E623 was computed along the trajectories based on each structure (Figure S3). Y730 was chosen over Y731 because Y731 is known to explore other conformations, whereas Y730 is much less mobile. In the simulations based on the midturnover structure (Figure S3B), E623 is indeed stable, remaining ∼8 Å away from Y730, comparable to its distance in the cryo-EM structure. However, in all three independent trajectories based on the preturnover structure (Figure S3A), E623 drifted away

from Y730 and Y731. These unbiased simulations cannot determine the relative free energies of the different conformations of E623, but they suggest that E623 may not always be in a position that enables it to mediate PCET between Y731 and Y730.[5,22] This motion of E623 was not observed in our previous work on the preturnover structure, which employed the older ff14SB Amber force field.[23]

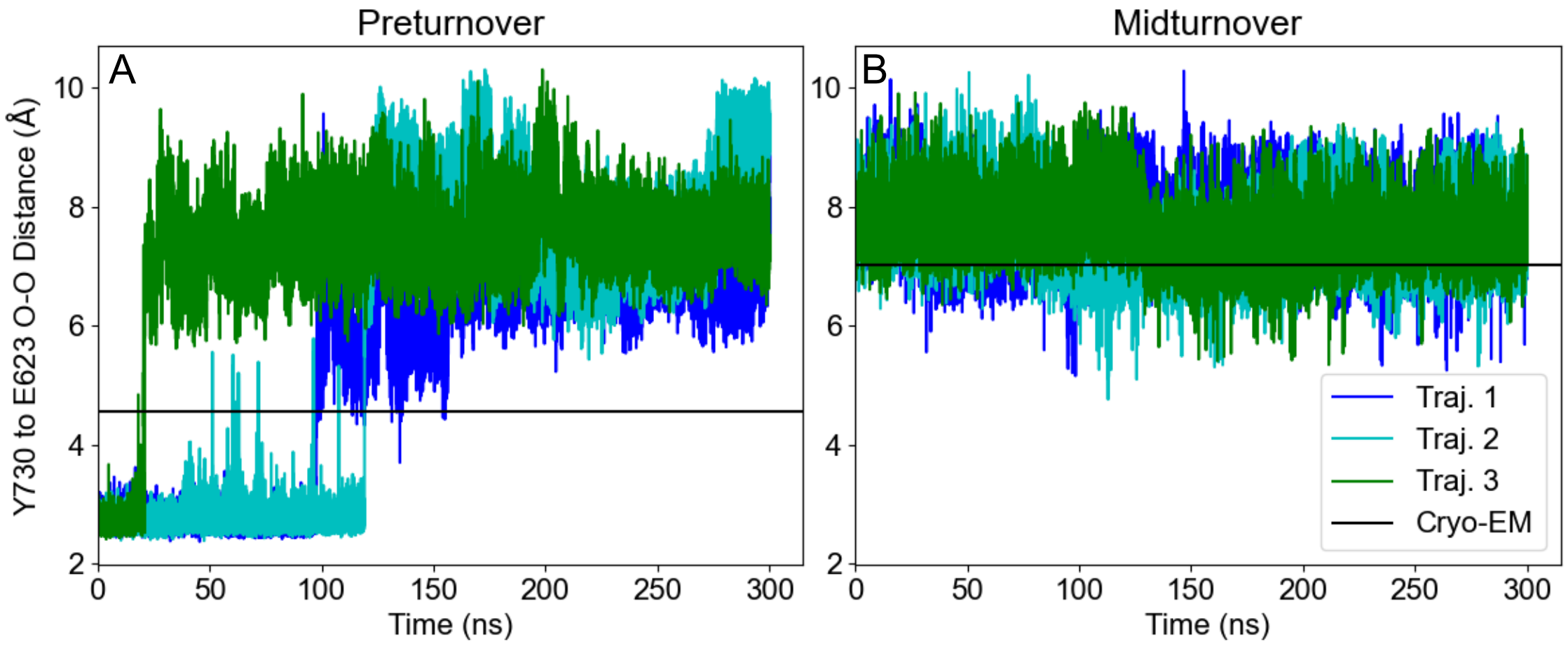

Figure S3: Distance between the phenoxyl oxygen of Y730 and the closer carboxylate oxygen of E623 in the production MD trajectories based on (A) the preturnover structure and (B) the midturnover structure. The three colors correspond to the three independent trajectories, which were each initiated from the same equilibrated structure with different initial velocities, and the black line corresponds to the distance observed in the respective cryo-EM structure.

The production trajectories shown in Figure S3A were initiated from the same equilibrated structure with different initial velocities. This aspect of the simulations raises the possibility that the equilibrated structure was from a region of conformational space in which this motion of E623 is favored, while the conformations sampled in our previous work were not. To address this possibility, we performed the entire equilibration procedure, including the energy minimizations and heating, three additional times, each starting from the preturnover cryo-EM structure. We then propagated three independent trajectories, where each one started from one of these three independently equilibrated structures. This procedure reduces the possibility that they will be trapped in the same region of conformational space. The C_α RMSDs for the three independent trajectories during the equilibration and production trajectories are shown in Figure S4A and indicate that each was adequately

equilibrated.

The distances from the phenoxyl oxygen of Y730 to the closer carboxylate oxygen of E623 during these three independent production trajectories are shown in Figure S4B, along with this distance in the cryo-EM structure for comparison. In two of the three trajectories, E623 moved away from Y730 during equilibration, while in the third trajectory, it remained close enough to form a hydrogen bond to Y730 throughout the 300 ns of production MD. These results indicate that E623 can sample many different conformations with the ff19SB force field. Enhanced sampling techniques could be used to estimate the relative free energies of the different conformations of E623, although identifying reaction coordinates to characterize these conformations may not be straightforward.

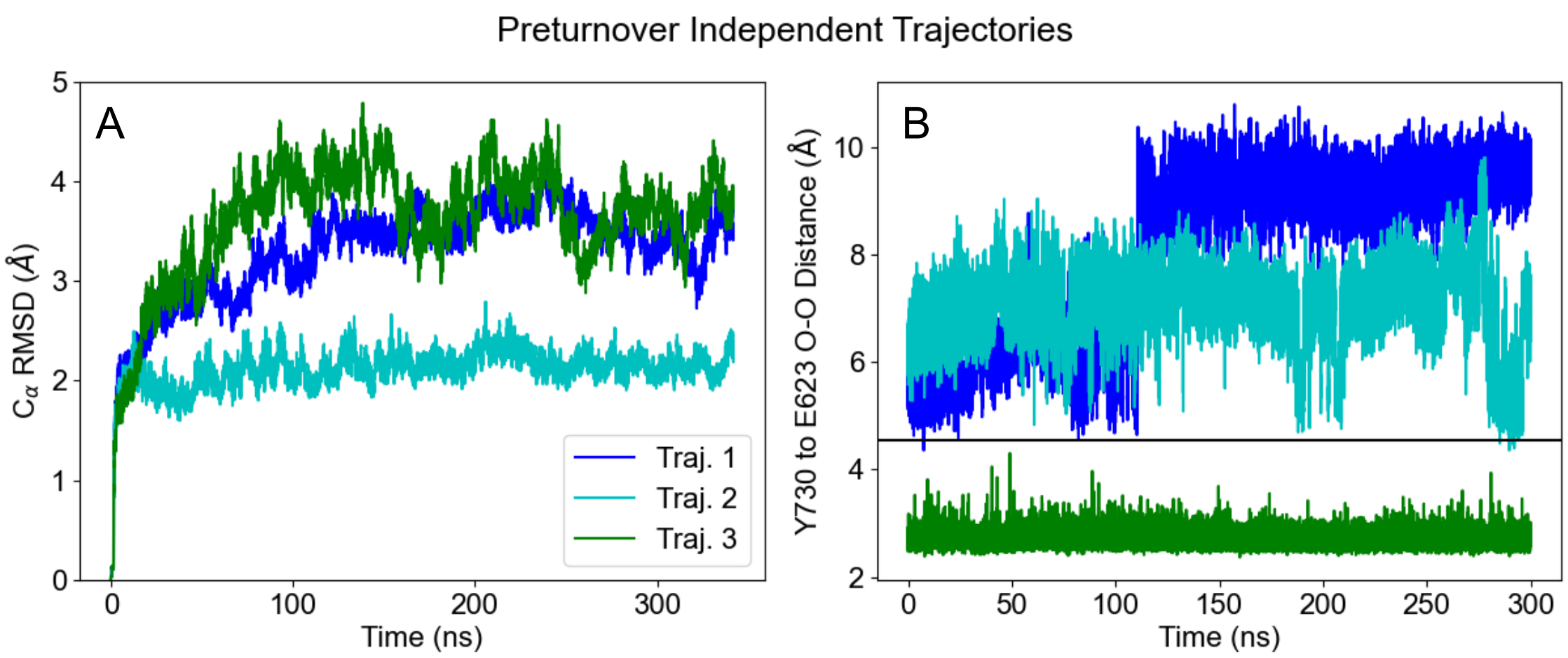

Figure S4: (A) C_α RMSD with respect to the preturnover cryo-EM structure during the equilibration and production MD trajectories. These data correspond to the three independently equilibrated trajectories starting from the preturnover cryo-EM structure. The equilibration phase ended at 40 ns. (B) Distance between the phenoxyl oxygen of Y730 and the closer carboxylate oxygen of E623 in these three independent production MD trajectories based on the preturnover structure. The three colors correspond to the three independent trajectories, where each one started from a structure independently equilibrated from the cryo-EM structure, and the black line in (B) corresponds to the distance observed in the cryo-EM structure.

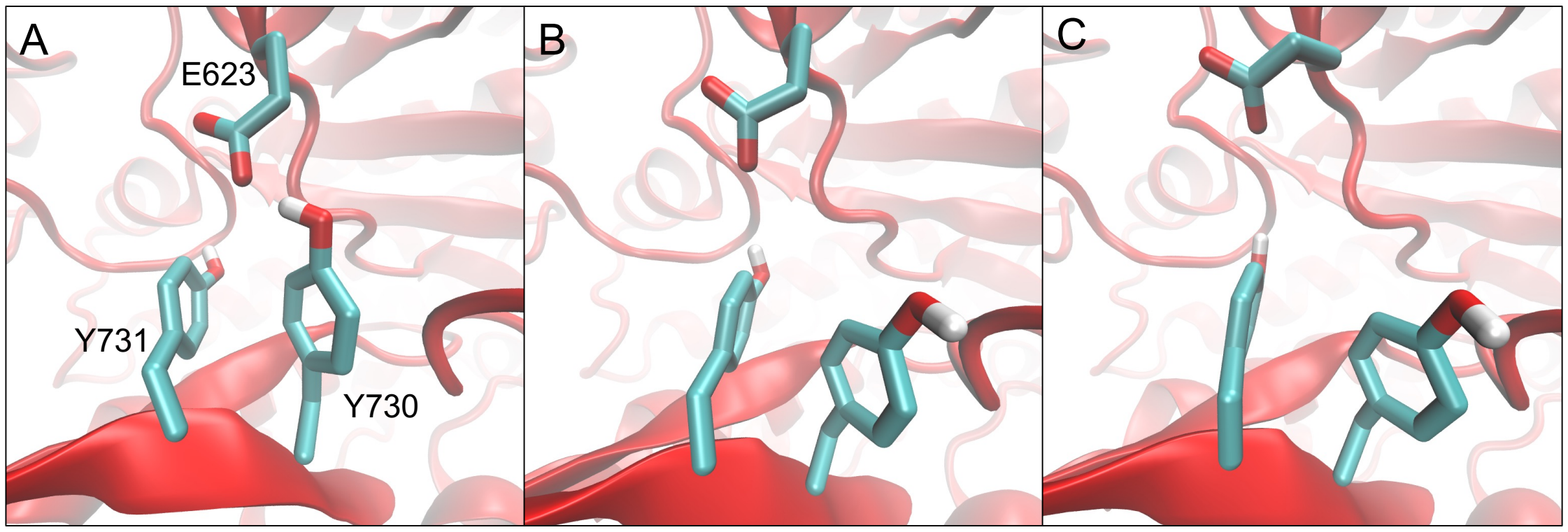

Figure S5: Representative conformations from a single MD trajectory based on the pre-turnover structure showing how E623 can reposition further from Y730. (A) E623 forms a hydrogen bond to Y730 during equilibration, which remains stable for a portion of the production trajectory. (B) The β-strand containing Y730 and Y731 can shift slightly away from E623, breaking the hydrogen bond. (C) Rotation about the C−C bonds in the flexible sidechain of E623 reorient its carboxylate group toward the α/β subunit subunit interface, where it forms the hydrogen-bonding network shown in Figure 4 of the main text. For visual clarity, only E623, Y731, Y730, and the backbone of the α subunit are shown.

5 QM/MM String Simulations

QM/MM finite temperature string simulations with umbrella sampling were used to generate the multidimensional free energy surfaces and find the minimum free energy path (MFEP) connecting the reactants and products for radical transfer from Y731 to Y730. As discussed in the main text, five different string simulations of this reaction were performed. First, we provide the computational details that apply to all of these simulations, and then we provide the details specific to each string.

After selection of a starting structure for the solvated protein system, any solvent (water molecule or ion) more than 3.5 Å from all solute atoms and more than 18 Å from the phenoxyl oxygen of Y731 (or 25 Å for the postturnover α' subunit string) was stripped away. This decrease in system size prevented the nonbonded pair list from becoming too large for Amber. Thus, these simulations were performed without periodic boundary conditions, and all nonbonded interactions were included. For one of the string simulations, the direct mechanism in the midturnover structure, an additional water molecule was stripped away near Y731 and Y730.

As in previous work,[22,24,25] the QM region was treated with the ωB97X-D functional[26] and 6-31+G** basis set.[27] This level of theory was found to be consistent with previous results obtained using multireference wavefunction methods.[22] The MM region was described by the force fields discussed in Section 1 of the Supporting Information. The link atoms between the QM and MM regions were added automatically by the Amber software. The Amber/Q-Chem 6.0[28] interface[29] was used for the energy minimizations and MD simulations. The initial velocities for the MD simulations were obtained from a Boltzmann distribution at 300 K, and the QM/MM MD simulations were performed in the NVE ensemble due to the short sampling for each iteration and randomization of velocities prior to each iteration. The harmonic restraints used for the umbrella sampling had initial values of 100 kcal/mol/Å^2, but this value was increased if needed for conformations that were more difficult to sample (*e.g.*, at the top of the free energy barrier).

Then, we removed the proton on the phenoxyl group of Y731 (or Y730) for the reactant (or product) structure to generate Y731$^{\bullet}$/Y730 (Y731/Y730$^{\bullet}$). The QM region included at least Y731 and Y730 (including their backbone) and, in some cases, also included a water molecule or the sidechain of E623, depending on the specific string, as discussed further below. We performed a QM/MM energy minimization for the initial reactant and product structures generated for each string. The optimized reactant structure was then optimized again using harmonic restraints to position the transferring proton(s) equidistant from the donor and acceptor oxygen atoms to approximate a transition state. During these QM/MM optimization steps, all atoms outside the QM region were frozen.

The initial coordinates for the 20 string images connecting the reactant and product were generated by quadratic interpolation of these initial reactant, approximate transition state, and product geometries. This interpolation yielded initial values of the equilibrium reaction coordinates for the harmonic restraints applied to these reaction coordinates for each image. The geometric reaction coordinates used for each string are defined below. For each image, the MM region was equilibrated using an MM force field for 10 ps to allow the environment

around the QM region relax, with the QM region and all residues more than 18 Å from the phenoxyl oxygen of Y731 frozen. During this MM equilibration, the atomic partial charges for the QM region were set to those of Y731$^{\bullet}$ and Y730, even if the geometries were more similar to Y731 and Y730$^{\bullet}$ for the later images in the string. As the charge redistribution involved in the net hydrogen atom transfer reaction is relatively small, this approximation is reasonable for this initial MM equilibration, which was followed by QM/MM equilibration using accurate charge distributions of the QM region. A timestep of 0.5 fs was used for this MM equilibration to avoid problems arising from unusual QM region geometries created by the interpolation. During the MM equilibration, the Andersen-like thermostat[30] was used to maintain a temperature of 300 K. From this step onward in the string simulations, all solute residues and solvent more than 18 Å from the phenoxyl oxygen of Y731 were frozen.

Next, each image was equilibrated with QM/MM MD for 100 fs, applying the harmonic restraints to the reaction coordinates associated with each image. These equilibrated structures were used to start the first iteration of the string procedure. For each iteration, 100 fs of QM/MM MD were propagated for each image. For each reaction coordinate, a fourth- or fifth-order polynomial was fit to the average values of that reaction coordinate over all images of the string. The images were then evenly distributed along this fitted string, providing values of the reaction coordinates used for the harmonic restraints for the next iteration. This procedure was repeated until the string converged. The binless variant of the WHAM algorithm was used to generate the multidimensional free energy surfaces utilizing the data generated from all iterations. The reaction coordinates for all images associated with the last iteration define the MFEP connecting the reactant and product.

A string was considered to be converged if the change in the reaction free energy and free energy barrier was less than 0.2 kcal/mol for the last five iterations and the RMSD of the reaction coordinates in the final iteration relative to their average over the last five iterations was less than 0.1 Å. In two cases (the through-water mechanism based on the midturnover structure and the direct mechanism based on the preturnover structure), the string began to

oscillate around the MFEP. However, the magnitude of the oscillations was sufficiently small (< 1 kcal/mol) such that the strings were considered converged. If the reactant and product regions were not adequately explored during the string procedure, the string was extended by adding images to the beginning or end by extrapolation and continuing with the string procedure.

For reactions involving a water molecule in the QM region, a three-point structure for that water molecule was created by deleting the dummy atom. Partial charges and bonded and nonbonded parameters for the OPC3[31] water model were assigned to this water molecule as default values. However, the point charges were only used during MM equilibration, and the bonded terms were never used because this molecule was either frozen (during MM equilibration) or treated quantum mechanically (during the minimizations and string iterations).

5.1 Direct — Midturnover

The initial structure for the string corresponding to the direct mechanism in simulations based on the midturnover structure was the conformation immediately following NVT equilibration. One water molecule that formed a hydrogen bond with Y731 and Y730 was removed prior to the initial QM/MM minimization to facilitate the direct mechanism. The QM region contained only Y731 and Y730, and the three reaction coordinates shown in Figure S6A were used. Convergence was obtained after 25 iterations of the string procedure. Figure S6 shows the reaction coordinates and the free energy profile along the MFEP for the converged string, as well as a projected two-dimensional (2D) free energy surface for the reaction.

5.2 Water-Mediated — Midturnover

The initial structure for the string corresponding to the water-mediated mechanism in simulations based on the midturnover structure was the same as that used for the direct mechanism,

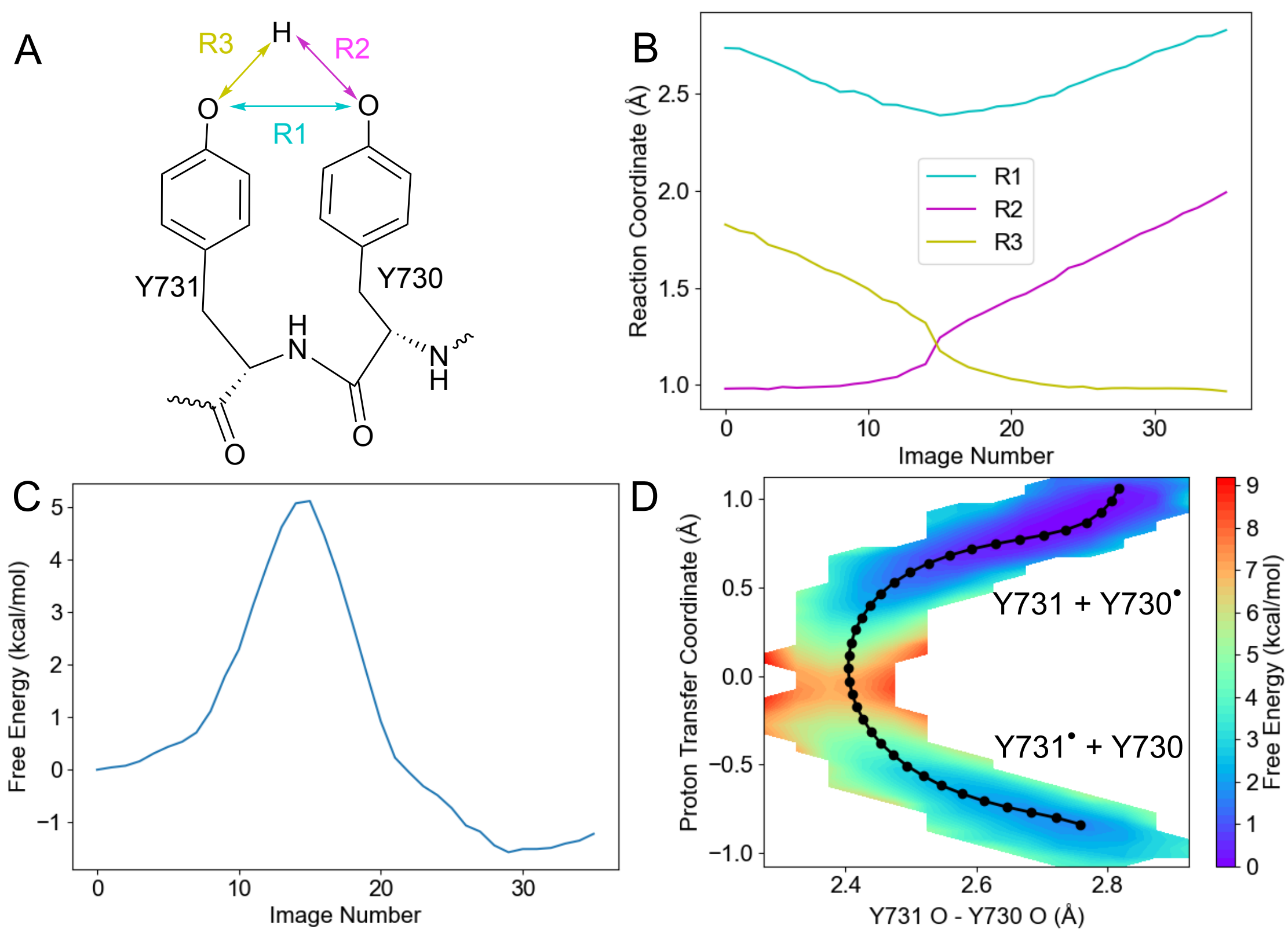

Figure S6: The direct mechanism for radical transfer from Y731 to Y730 in string simulations based on the midturnover structure. (A) The QM region and reaction coordinates used to characterize this reaction. (B) The average values of the three reaction coordinates along the converged string. (C) The free energy profile along the MFEP for the converged string. (D) The free energy surface of this reaction as a function of the distance between the phenoxyl oxygen atoms of Y731 and Y730 and the proton transfer coordinate, defined as R2 − R3 such that negative values correspond to the reactant and positive values correspond to the product. The MFEP for the converged string is shown in black.

but the hydrogen-bonded water was not removed. The QM region contained Y731, Y730, and the water molecule mediating the reaction. The five reaction coordinates are shown in Figure S7A. Convergence was obtained after 46 iterations of the string procedure. Figure S7 shows the reaction coordinates and the free energy profile along the MFEP for the converged string, as well as a projected 2D free energy surface for the reaction.

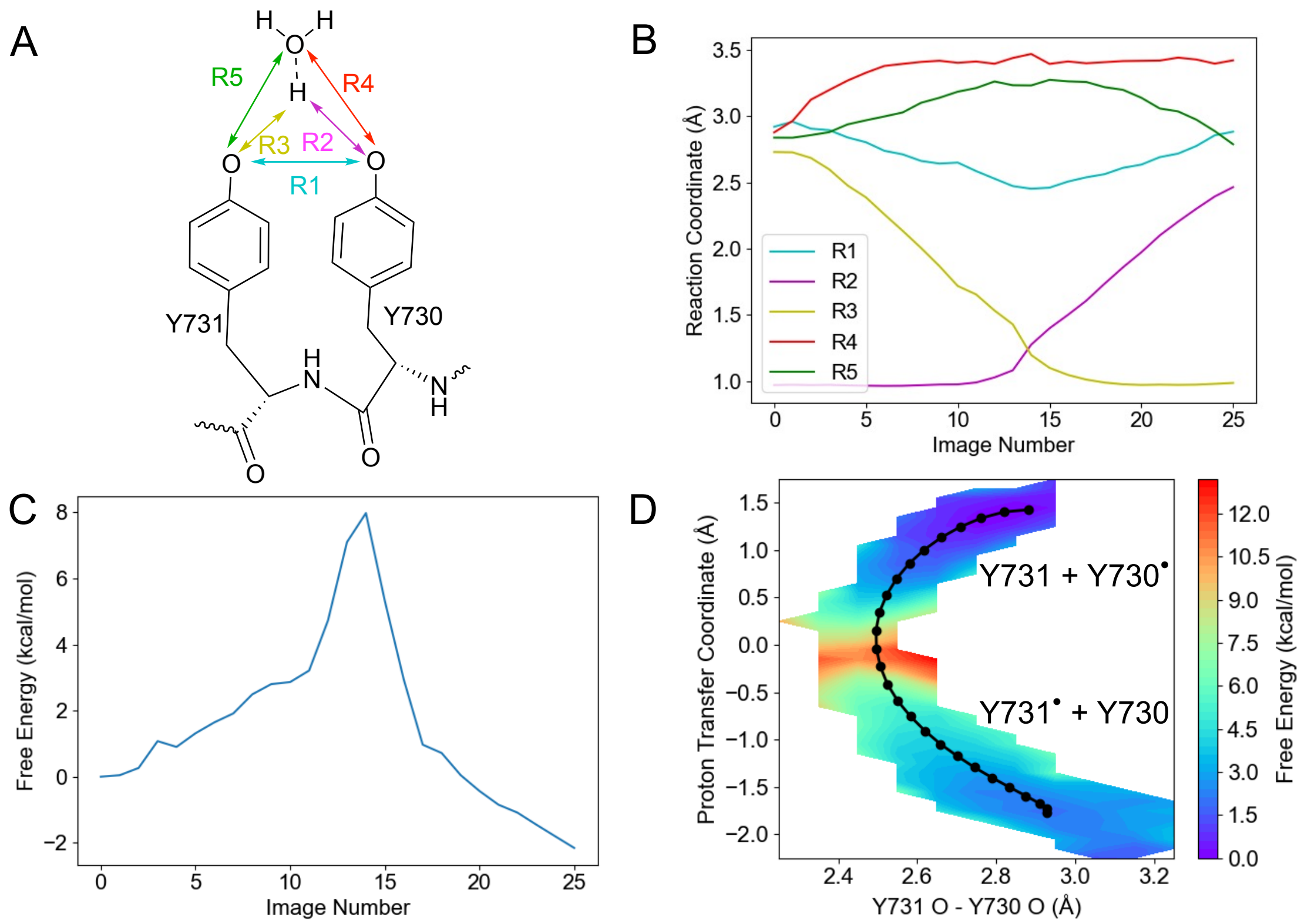

Figure S7: The water-mediated mechanism for radical transfer from Y731 to Y730 in string simulations based on the midturnover structure. (A) The QM region and reaction coordinates used to characterize this reaction. (B) The average values of the five reaction coordinates along the converged string. (C) The free energy profile along the MFEP for the converged string. (D) The free energy surface of this reaction as a function of the distance between the phenoxyl oxygen atoms of Y731 and Y730 and the proton transfer coordinate, defined as R2 − R3 such that negative values correspond to the reactant and positive values correspond to the product. The MFEP for the converged string is shown in black.

5.3 Through-Water — Midturnover

The initial structure for the string corresponding to the through-water mechanism in simulations based on the midturnover structure was the same as that used for the direct mechanism, but the hydrogen-bonded water was not removed. During the QM/MM minimization steps, harmonic restraints were applied to create the reactant, product, and "transition state" geometries. In the reactant, Y730 donates a hydrogen bond to the mediating water molecule, which donates a hydrogen bond to Y731$^{\bullet}$. In the product, both protons have transferred, and Y731 donates a hydrogen bond to the mediating water molecule, which donates a hydrogen bond to Y730$^{\bullet}$. In the "transition state," the transferring protons were restrained to be equidistant from their respective donor and acceptor oxygen atoms. The QM region contained Y731, Y730, and the water molecule mediating the reaction. The six reaction coordinates are shown in Figure S8A. After 26 iterations of the string procedure, it was observed that the relative free energy and free energy barrier of the reaction oscillated, with a change in magnitude of < 1 kcal/mol. At this point, the string was considered to be sufficiently converged for our analysis. Figure S8 shows the free energy profile and the reaction coordinates along the MFEP for the converged string.

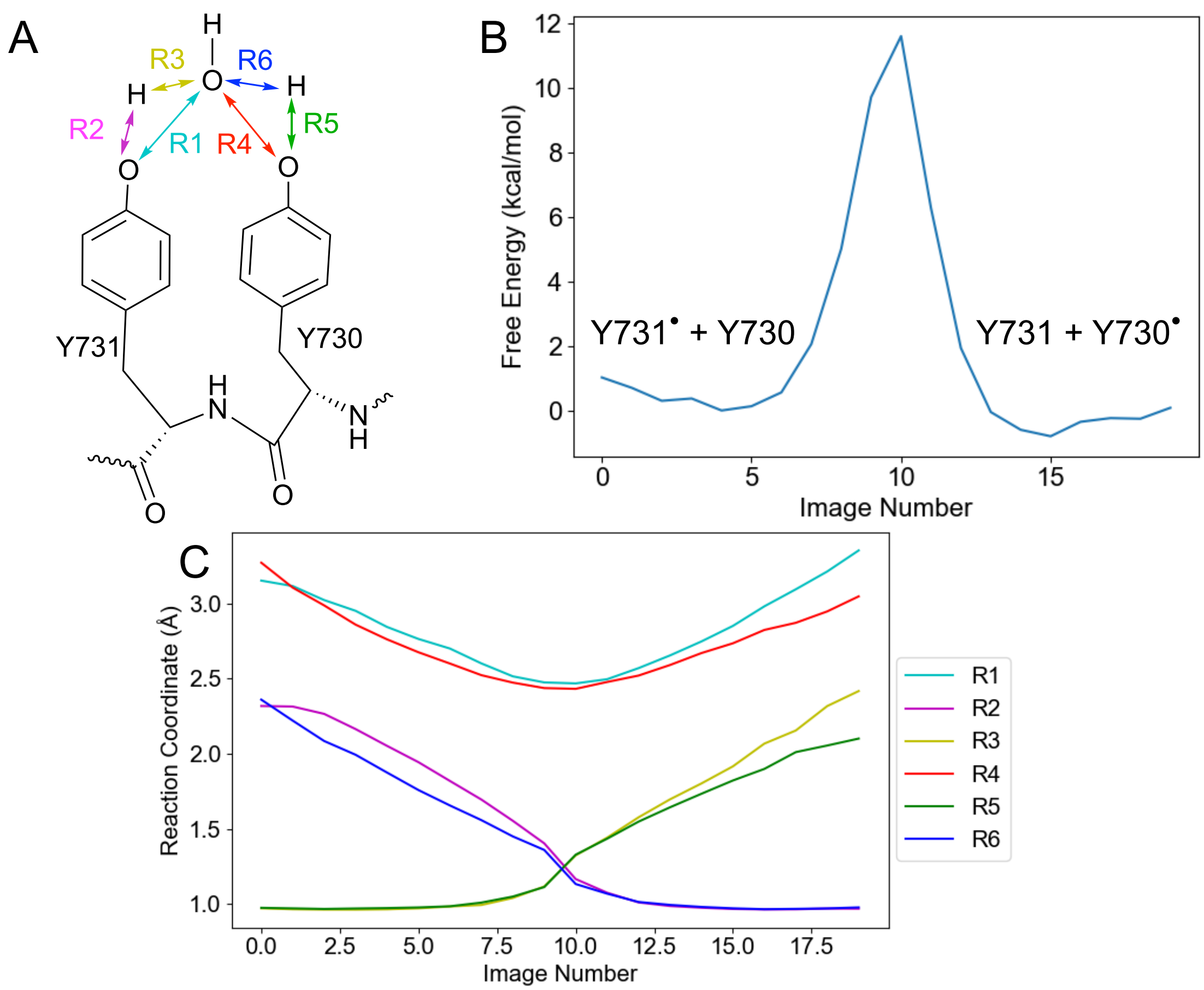

Figure S8: The through-water mechanism for radical transfer from Y731 to Y730 in string simulations based on the midturnover structure. (A) The QM region and reaction coordinates used to characterize this reaction. (B) The free energy profile along the MFEP for the converged string. (C) The average values of the six reaction coordinates along the MFEP for the converged string.

5.4 Postturnover α' — Midturnover

The initial structure for the string corresponding to the α' subunit in simulations based on the midturnover structure was a conformation from the production trajectory in which the χ_1 dihedral angle of Y731 in the α' subunit was $\geq 300°$. The QM region contained only Y730 and Y731. The three reaction coordinates are shown in Figure S9A. Convergence of the string procedure was obtained after 64 iterations. Figure S9 shows the reaction coordinates and the free energy profile along the MFEP for the converged string, as well as a projected 2D free energy surface for the reaction.

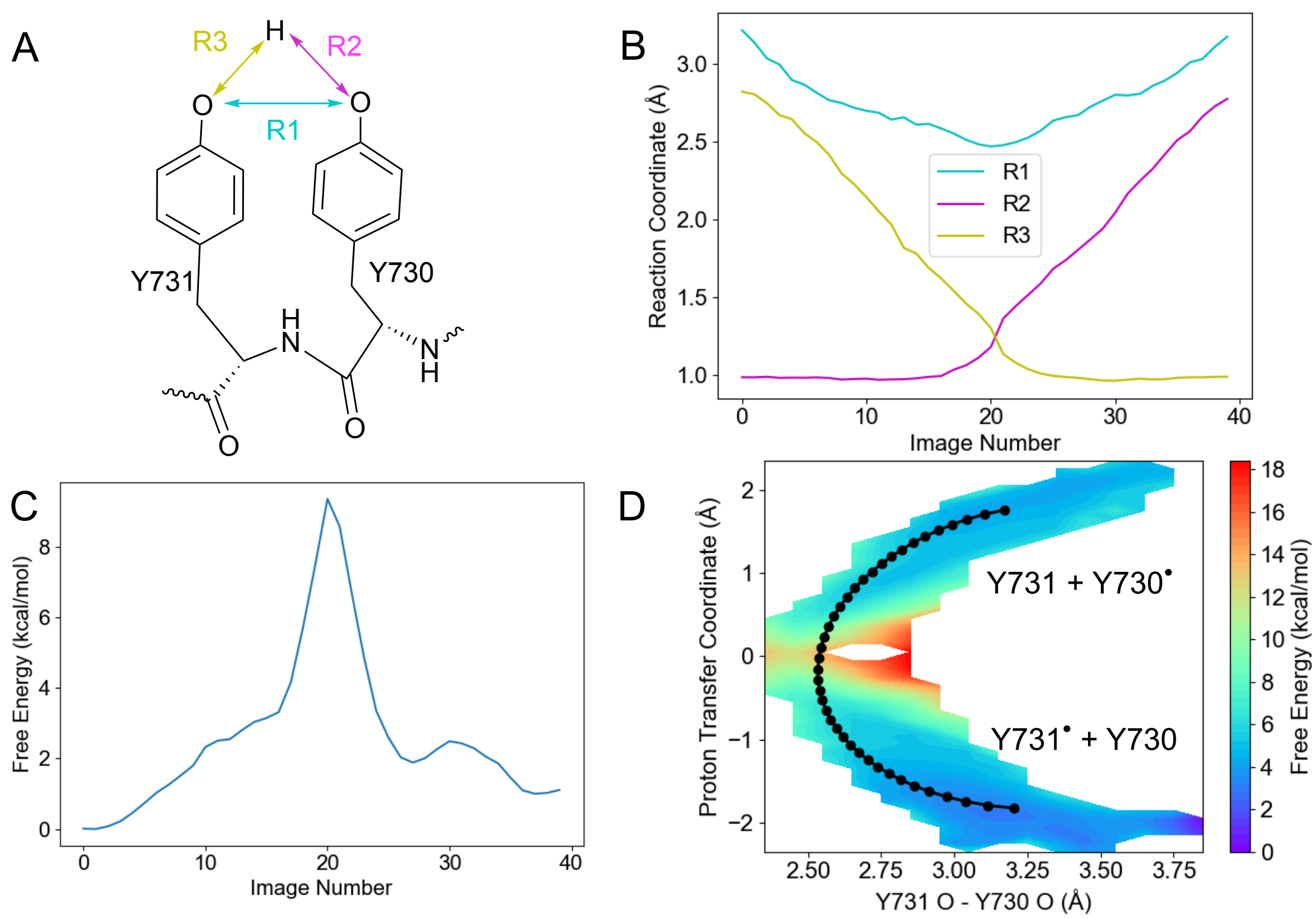

Figure S9: The direct mechanism for radical transfer from Y731 to Y730 in the postturnover α' subunit in string simulations based on the midturnover structure. (A) The QM region and reaction coordinates used to characterize this reaction. (B) The average values of the three reaction coordinates along the converged string. (C) The free energy profile along the MFEP for the converged string. (D) The free energy surface of this reaction as a function of the distance between the phenoxyl oxygen atoms of Y731 and Y730 and the proton transfer coordinate, defined as R2 − R3 such that negative values correspond to the reactant and positive values correspond to the product. The MFEP for the converged string is shown in black.

5.5 Direct — Preturnover

The initial structure for the string corresponding to the direct mechanism in simulations based on the preturnover structure was a conformation from the preturnover production trajectory in which the Y731 χ_1 dihedral angle was between 270 and 330° and each of the carboxylate oxygen atoms of E623 was at least 6 Å from the phenoxyl oxygen atom of Y730. In this conformation, Y730 and Y731 are stacked, and E623 is sufficiently far from them such that it is unable to mediate the PCET reaction. The QM region contained only Y731 and Y730. During the QM/MM minimization steps for the reactant, approximate transition state, and product geometries, harmonic restraints were applied to the reaction coordinates based on their values in the converged string from our previous study of the PCET reaction between Y731 and Y730 in this structure.[22] After 43 iterations of the string procedure, it was observed that the relative free energy and free energy barrier of the reaction oscillated, with a change in magnitude of < 1 kcal/mol. At this point, the string was considered to be sufficiently converged for our analysis. Figure S10 shows the reaction coordinates and the free energy profile along the MFEP for the converged string, as well as a projected 2D free energy surface for the reaction.

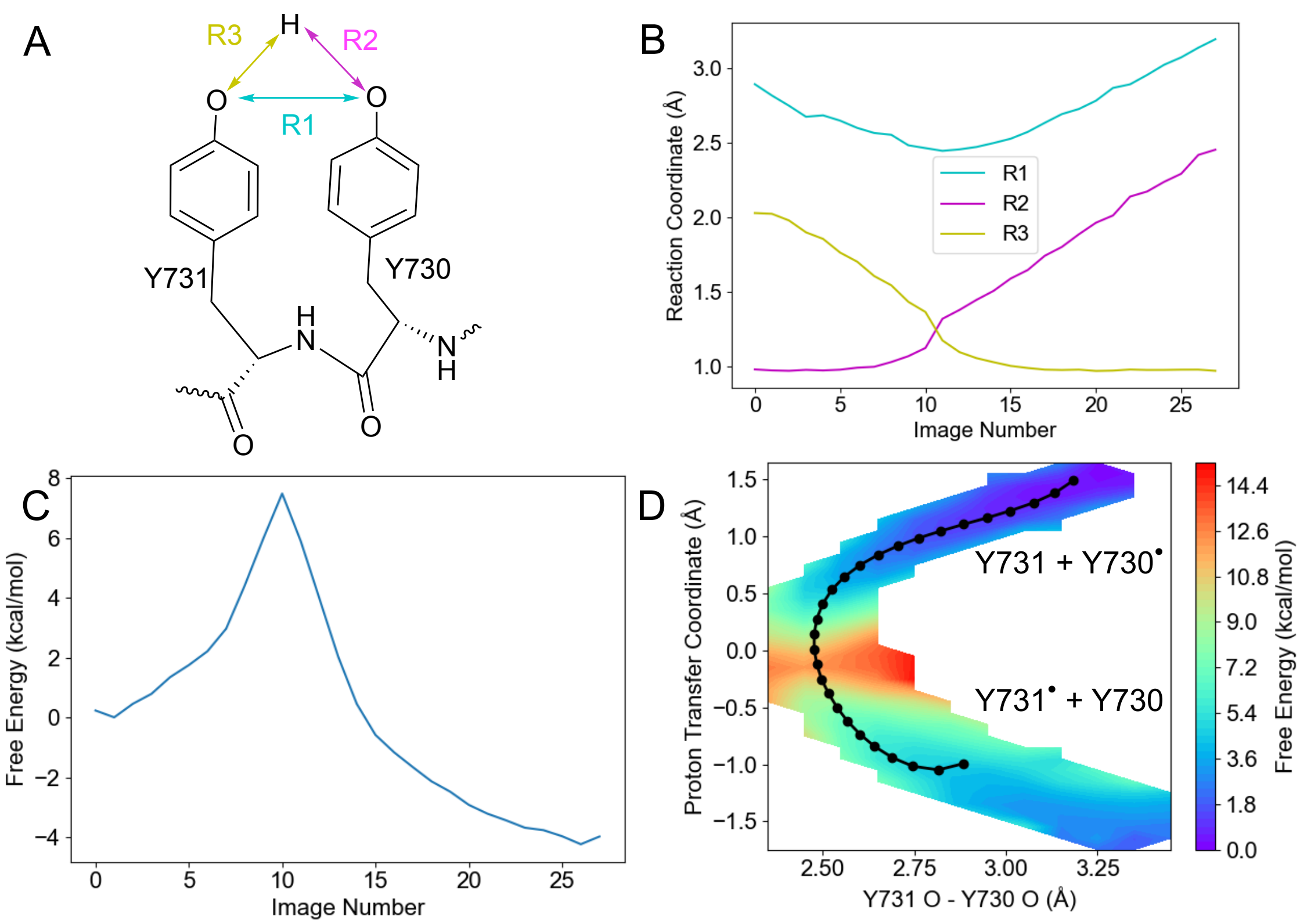

Figure S10: The direct mechanism for radical transfer from Y731 to Y730 in string simulations based on the preturnover structure. (A) The QM region and reaction coordinates used to characterize this reaction. (B) The average values of the three reaction coordinates along the converged string. (C) The free energy profile along the MFEP for the converged string. (D) The free energy surface of this reaction as a function of the distance between the phenoxyl oxygen atoms of Y731 and Y730 and the proton transfer coordinate, defined as R2 − R3 such that negative values correspond to the reactant and positive values correspond to the product. The MFEP for the converged string is shown in black.

5.6 Convergence and Statistical Analyses

The free energy profiles for the final string iteration and the five iterations before that iteration for each string simulation are shown in Figure S11. To estimate the statistical uncertainty in the sampling, bootstrapping was performed by resampling to create 100 bootstrapped free energy profiles. Figure S12 shows the mean free energy profile from these simulations with the standard deviation as error bars. Note that bootstrapping only provides information on statistical errors and does not account for underlying errors arising from the the level of theory used and the limited sampling of much slower conformational

motions.

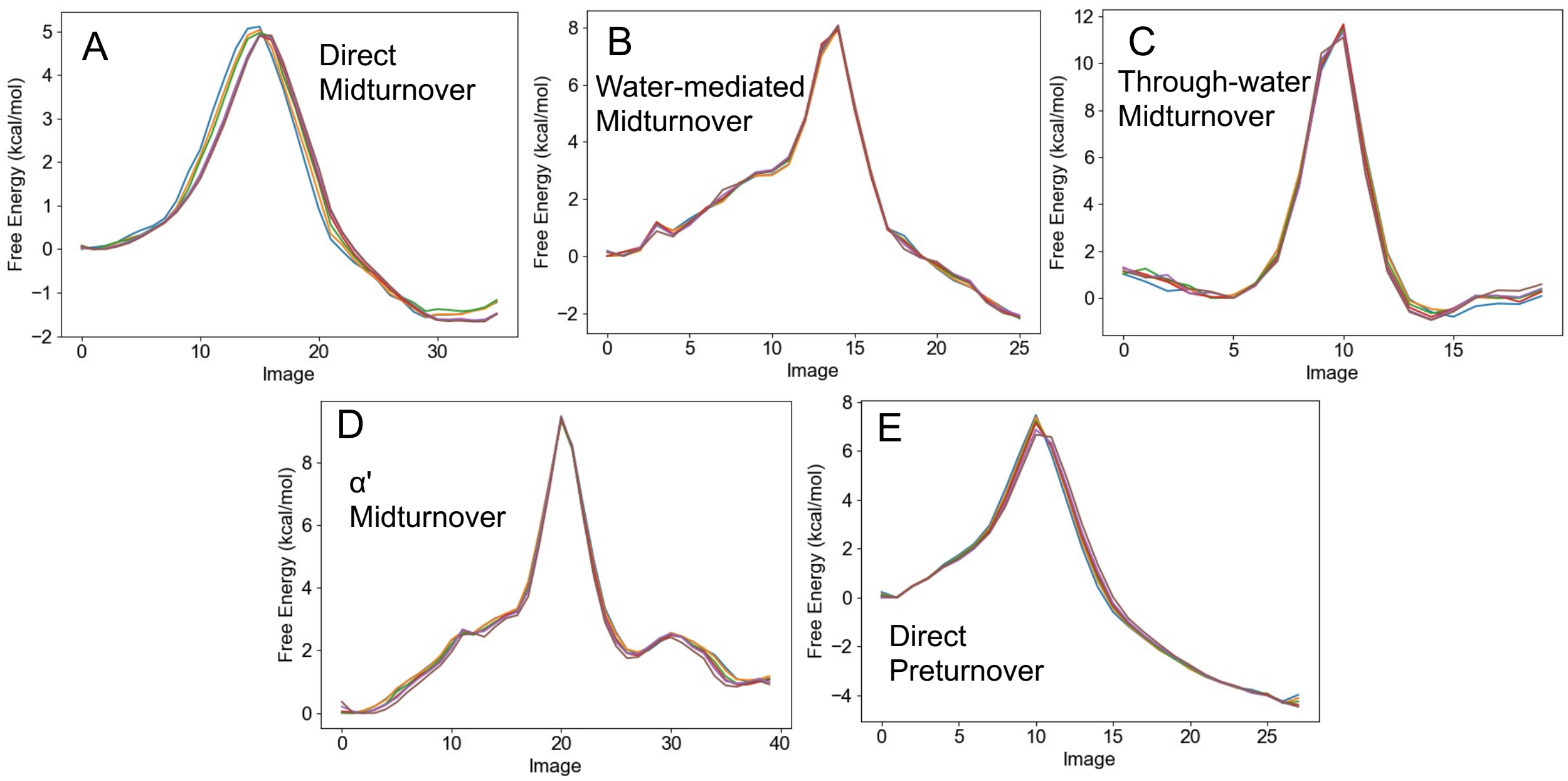

Figure S11: The free energy profiles corresponding to the final string iteration and the previous five iterations for each of the five reactions considered in this work. The data are shown for the following strings: (A) direct mechanism based on the midturnover structure, (B) water-mediated mechanism based on the midturnover structure, (C) through-water mechanism based on the midturnover structure, (D) direct mechanism in the α' subunit based on the midturnover structure, and (E) direct mechanism based on the preturnover structure.

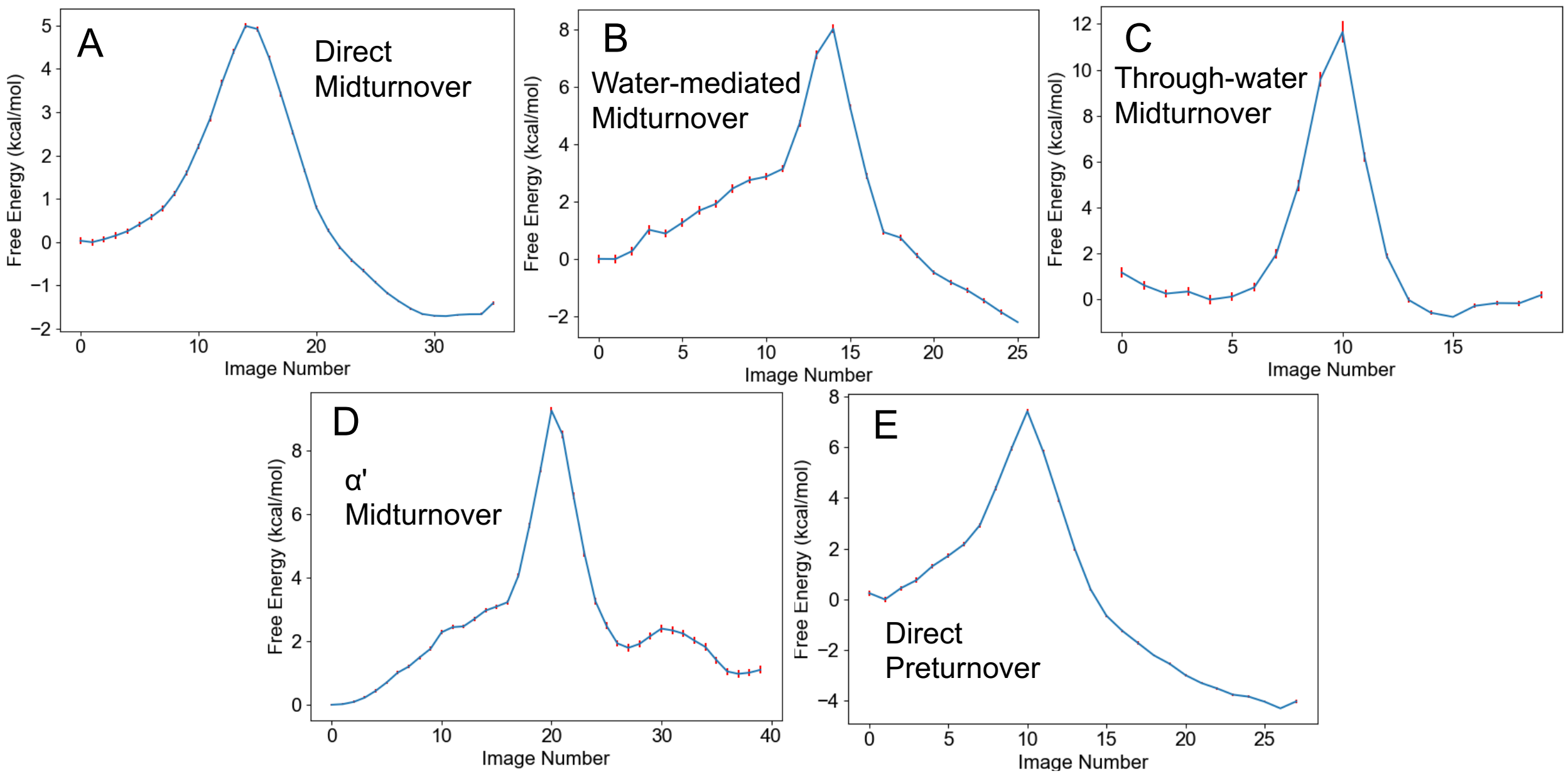

Figure S12: The bootstrapped free energy profiles corresponding to the final string iteration and the previous five iterations for each of the five reactions considered in this work. The mean free energy profile is shown in blue, and the red error bars represent the standard deviation from bootstrapping. The data are shown for the following strings: (A) direct mechanism based on the midturnover structure, (B) water-mediated mechanism based on the midturnover structure, (C) through-water mechanism based on the midturnover structure, (D) direct mechanism in the α' subunit based on the midturnover structure, and (E) direct mechanism based on the preturnover structure.

References

(1) Case, D. A. et al. AmberTools. *J. Chem. Inf. Model.* **2023**, *63*, 6183–6191.

(2) Gordon, J. C.; Myers, J. B.; Folta, T.; Shoja, V.; Heath, L. S.; Onufriev, A. H++: a server for estimating p Ka s and adding missing hydrogens to macromolecules. *Nucleic Acids Res.* **2005**, *33*, W368–W371.

(3) Izadi, S.; Anandakrishnan, R.; Onufriev, A. V. Building Water Models: A Different Approach. *J. Phys. Chem. Lett.* **2014**, *5*, 3863–3871.

(4) Sengupta, A.; Li, Z.; Song, L. F.; Li, P.; Merz, K. M. J. Parameterization of Monovalent

Ions for the OPC3, OPC, TIP3P-FB, and TIP4P-FB Water Models. *J. Chem. Inf. Model.* **2021**, *61*, 869–880.

(5) Reinhardt, C. R.; Li, P.; Kang, G.; Stubbe, J.; Drennan, C. L.; Hammes-Schiffer, S. Conformational Motions and Water Networks at the α/β Interface in E. coli Ribonucleotide Reductase. *J. Am. Chem. Soc.* **2020**, *142*, 13768–13778.

(6) Tian, C.; Kasavajhala, K.; Belfon, K. A. A.; Raguette, L.; Huang, H.; Migues, A. N.; Bickel, J.; Wang, Y.; Pincay, J.; Wu, Q.; Simmerling, C. ff19SB: Amino-Acid-Specific Protein Backbone Parameters Trained against Quantum Mechanics Energy Surfaces in Solution. *J. Chem. Theory Comput.* **2020**, *16*, 528–552.

(7) Jorgensen, W. L.; Chandrasekhar, J.; Madura, J. D.; Impey, R. W.; Klein, M. L. Comparison of simple potential functions for simulating liquid water. *J. Chem. Phys.* **1983**, *79*, 926–935.

(8) Kang, G.; Taguchi, A. T.; Stubbe, J.; Drennan, C. L. Structure of a trapped radical transfer pathway within a ribonucleotide reductase holocomplex. *Science* **2020**, *368*, 424–427.

(9) Westmoreland, D. E.; Feliciano, P. R.; Kang, G.; Cui, C.; Kim, A.; Stubbe, J.; Nocera, D. G.; Drennan, C. L. 2.6-Å resolution cryo-EM structure of a class Ia ribonucleotide reductase trapped with mechanism-based inhibitor N3CDP. *Proc. Natl. Acad. Sci. U.S.A.* **2024**, *121*, e2417157121.

(10) Bayly, C. I.; Cieplak, P.; Cornell, W.; Kollman, P. A. A well-behaved electrostatic potential based method using charge restraints for deriving atomic charges: the RESP model. *J. Phys. Chem.* **1993**, *97*, 10269–10280.

(11) Dennington, R.; Keith, T. A.; Millam, J. M. GaussView Version 6. 2019.

(12) Cieplak, P.; Cornell, W. D.; Bayly, C.; Kollman, P. A. Application of the multimolecule and multiconformational RESP methodology to biopolymers: Charge derivation for DNA, RNA, and proteins. *J. Comp. Chem.* **1995**, *16*, 1357–1377.

(13) Frisch, M. J. et al. Gaussian 16 Revision C.01. 2016.

(14) Meagher, K. L.; Redman, L. T.; Carlson, H. A. Development of polyphosphate parameters for use with the AMBER force field. *J. Comp. Chem.* **2003**, *24*, 1016–1025.

(15) Ivani, I. et al. Parmbsc1: a refined force field for DNA simulations. *Nat. Methods* **2016**, *13*, 55–58.

(16) Case, D. A. et al. Recent Developments in Amber Biomolecular Simulations. *J. Chem. Inf. Model.* **2025**, *65*, 7835–7843.

(17) Miyamoto, S.; Kollman, P. A. Settle: An analytical version of the SHAKE and RATTLE algorithm for rigid water models. *J. Comp. Chem.* **1992**, *13*, 952–962.

(18) Ryckaert, J.-P.; Ciccotti, G.; Berendsen, H. J. C. Numerical integration of the cartesian equations of motion of a system with constraints: molecular dynamics of *n*-alkanes. *J. Comp. Phys.* **1977**, *23*, 327–341.

(19) Darden, T.; York, D.; Pedersen, L. Particle mesh Ewald: An Nlog(N) method for Ewald sums in large systems. *J. Chem. Phys.* **1993**, *98*, 10089–10092.

(20) Grossfield, A. WHAM: an implementation of the weighted histogram analysis method, version 2.1.0. 2000.

(21) Kumar, S.; Rosenberg, J. M.; Bouzida, D.; Swendsen, R. H.; Kollman, P. A. THE weighted histogram analysis method for free-energy calculations on biomolecules. I. The method. *J. Comp. Chem.* **1992**, *13*, 1011–1021.

(22) Reinhardt, C. R.; Sayfutyarova, E. R.; Zhong, J.; Hammes-Schiffer, S. Glutamate Mediates Proton-Coupled Electron Transfer Between Tyrosines 730 and 731 in Escherichia coli Ribonucleotide Reductase. *J. Am. Chem. Soc.* **2021**, *143*, 6054–6059.

(23) Maier, J. A.; Martinez, C.; Kasavajhala, K.; Wickstrom, L.; Hauser, K. E.; Simmerling, C. ff14SB: Improving the Accuracy of Protein Side Chain and Backbone Parameters from ff99SB. *J. Chem. Theory Comput.* **2015**, *11*, 3696–3713.

(24) Zhong, J.; Reinhardt, C. R.; Hammes-Schiffer, S. Role of Water in Proton-Coupled Electron Transfer between Tyrosine and Cysteine in Ribonucleotide Reductase. *J. Am. Chem. Soc.* **2022**, *144*, 7208–7214.

(25) Zhong, J.; Reinhardt, C. R.; Hammes-Schiffer, S. Direct Proton-Coupled Electron Transfer between Interfacial Tyrosines in Ribonucleotide Reductase. *J. Am. Chem. Soc.* **2023**, *145*, 4784–4790.

(26) Chai, J.-D.; Head-Gordon, M. Long-range corrected hybrid density functionals with damped atom–atom dispersion corrections. *Phys. Chem. Chem. Phys.* **2008**, *10*, 6615.

(27) Hehre, W. J.; Ditchfield, R.; Pople, J. A. Self—Consistent Molecular Orbital Methods. XII. Further Extensions of Gaussian—Type Basis Sets for Use in Molecular Orbital Studies of Organic Molecules. *J. Chem. Phys.* **1972**, *56*, 2257–2261.

(28) Epifanovsky, E. et al. Software for the frontiers of quantum chemistry: An overview of developments in the Q-Chem 5 package. *J. Chem. Phys.* **2021**, *155*, 084801.

(29) Götz, A. W.; Clark, M. A.; Walker, R. C. An extensible interface for QM/MM molecular dynamics simulations with AMBER. *J. Comp. Chem.* **2014**, *35*, 95–108.

(30) Andrea, T. A.; Swope, W. C.; Andersen, H. C. The role of long ranged forces in determining the structure and properties of liquid water. *J. Chem. Phys.* **1983**, *79*, 4576–4584.

(31) Izadi, S.; Onufriev, A. V. Accuracy limit of rigid 3-point water models. *J. Chem. Phys.* **2016**, *145*, 074501.
